# Zooming in and out of semantics: proximal–distal construal levels and prominence hierarchies

**DOI:** 10.3389/fpsyg.2024.1371538

**Published:** 2024-09-09

**Authors:** Marit Lobben, Bruno Laeng

**Affiliations:** Department of Psychology, University of Oslo, Oslo, Norway

**Keywords:** prominence hierarchies, construal level theory (CLT), split ergativity, differential object marking (DOM), nominal classification, culture-language interpretive matrix, abstraction processes, animacy hierarchy

## Abstract

We argue that the “Prominence Hierarchy” within linguistics can be subsumed under the “Construal Level Theory” within psychology and that a wide spectrum of grammatical phenomena, ranging from case assignment to number, definiteness, verbal agreement, voice, direct/inverse morphology, and syntactic word-order respond to Prominence Hierarchies (PH), or semantic scales. In fact, the field of prominence hierarchies, as expressed through the languages of the world, continues to be riddled with riddles. We identify a set of conundrums: (A) vantage point and animacy, (B) individuation and narrow reference phenomena, (C) fronting mechanisms, (D) abstraction, and (E) cultural variance and flexibility. We here propose an account for the existence of these hierarchies and their pervasive effects on grammar by relying on psychological Construal Level Theory (CLT). We suggest that both PH and CLT structure the external world according to proximity or distance from the “*Me*, *Here* and *Now*” (MHN) perspective. In language, MHN has the effect of structuring grammars; in cognition, it structures our lives, our preferences, and choices.

## Introduction

We propose a domain-general explanation for linguistic prominence effects by merging two independent research traditions that started independently, about 50 years ago, within psychology (Schmitt, 1972; Vinokur et al., 1975; Trope and Burnstein, 1975) and linguistics (Silverstein, 1976). These fields of research have remained apparently oblivious to another, although—as we shall argue—they do relate to similar focus areas: Decision making within the field of social psychology, and a grammatical hierarchy known as “animacy hierarchies,” displayed in various structures of transitive sentences in highly unrelated languages.

We note that human decisions are often made in the moment, by an agent from the perspective of an ego, as well as with perspectives that an event may take place in the future. Also, according to linguistics, actions emanate in the unmarked case from a single individual and have consequences beyond the originator’s close sphere and further away in time–space. In both cases, the protagonist foresees a mental timeline. The distances traversed may differ in the two cases, but the trajectories are essentially the same, with an origin near a cognizing individual and an end state at some distance from this point, with wide-reaching effects on cognition. At close range, details, means, emotions, peers, time, and place matter. Immersed in a context, a subject cannot see the situation without bias. Transitory emotions are in focus rather than far-reaching often abstract, goals. From a distance, by contrast, the individual can view the essential similarities and differences.

Social psychologists have acknowledged the above aspects in a formal theory on mental construal levels, based on a wide array of empirical experiments (Soderberg et al., 2015). Linguists too, are on their way to understanding that the *prominence* that these grammatical hierarchies spring out from, has to do with an egocentric viewpoint, and not primarily, as often believed, with animacy and other *manifestations* of the hierarchy (Gardelle and Sorlin, 2018). Bridging the gap between linguistics and cognition is in line with a fundamental stance that whatever takes place in mental space is likely to influence the structure of language, in line with the fundamental tenets of cognitive linguistics (Tsoneva-Mathewson, 2009, p. 346):

“Cognitive linguistics encompasses a number of broadly compatible theoretical approaches to linguistic meaning and structure that share a common basis: the idea that language is an integral part of cognition, and it reflects the interaction of cultural, psychological, and communicative factors which can only be understood in the context of a realistic view of conceptualization and mental processing.”

Our argument runs as follows: We first attempt to explain the interdependence between markedness and prototypicality and how this relationship gives rise to prominence hierarchies. We then describe how cross-linguistic variance of seemingly arbitrary borderlines in category structure, as well as cut-off points in grammatical hierarchies, behave as gradient phenomena, and thus formulate a hypothesis based on Construal Level Theory (CLT) that specifies converging aspects of the two fields. From this point onwards, we go on to formulate a set of unsolved questions in prominence phenomena, subsumed under the label “conundrums.” To replenish the identified gaps, we concurrently present empirical support for our analysis and propose how prominence phenomena and cross-linguistic differences in category structure can be explained in terms of “psychological distance” within CLT. Finally, we summarize our findings and discuss briefly how our proposal intersects with previous linguistic analyses, as well as formulating predictions of our proposal as cases for future research.

### Markedness and prototypes

Since prototypical categorization (Rosch, 1978) is based on human experience and cognition rather than objective, mind-independent criteria (Geeraerts, 2016), it becomes ubiquitous in language (Taylor, 2003). Note also that prototypical category membership is not binary but exist on a continuum. The co-existence of peripheral and central members is critical to linguistic systems since it permits a high degree of flexibility for cognitive development. While peripheral members possess less of the features and properties associated with the category, prototypical members constitute a core which exhibit the most characteristic features. Importantly, these prototypes serve as cognitive reference points, or anchors, against which new potential category members are compared (van der Auwera and Gast, 2010). As people make new experiences, their mental representations of categories can shift, and prototypical structures evolve over time by means of overlap in features, metaphorical extension, and influence from technological or social changes. Semantic category structure may therefore also eventually be influenced by cultural mindsets (Aikhenvald, 2000, pp. 347, 421).

Prototypicality and markedness are two sides of the same coin. Markedness is what deviates from the prototypical (Croft, 1990, pp. 124–154) or the state of standing out as nontypical or divergent, as opposed to the regular or common. To define markedness, it then becomes paramount to identify the prototypical elements in language. This is done by observing inequalities in structural, behavioral, and frequency data (Greenberg, 1966), both cross-linguistically and within languages. In linguistic marked–unmarked relations, one term of an opposition is the broader, dominant, or typical one (known as *unmarked*); the other one is *marked* and may involve extra morphology or more complex semantics. For example, in the morphosyntactic category of Number, the singular is normally unmarked whereas the plural is marked, since the notion of plurality arises out of adding semantics and morphology to a less complex item. It is this asymmetry aspect that conceptually links markedness to the multivalued categories in implicational universals and grammatical categories: The essential idea behind markedness in typology is the “asymmetric or unequal grammatical properties of otherwise equal linguistic elements – inflections, […] or even syntactic constructions” (Croft, 1990, p. 64).

Markedness may be cancelled in particular contexts (Battistella, 1996, pp. 37, 144), in which the untypical becomes the typical. This is what is known as *markedness reversal* (Aissen, 1999, pp. 679–680) or local markedness (Croft, 1990, p. 66). Such reversals are affected by extralinguistic factors. For example, relative to plurals, singulars are marked in mass nouns (e.g., *oats*, *salt*) and with items typically occurring in pairs (e.g., *paired body parts*) (Tiersma, 1982). Croft (1990, p. 66) explains this behavior as “objects that naturally occur together or are difficult to individuate,” a way of thinking pertinent to the definition we adopt for prototypical transitivity.

### Markedness in sentences

**Figure 1:**
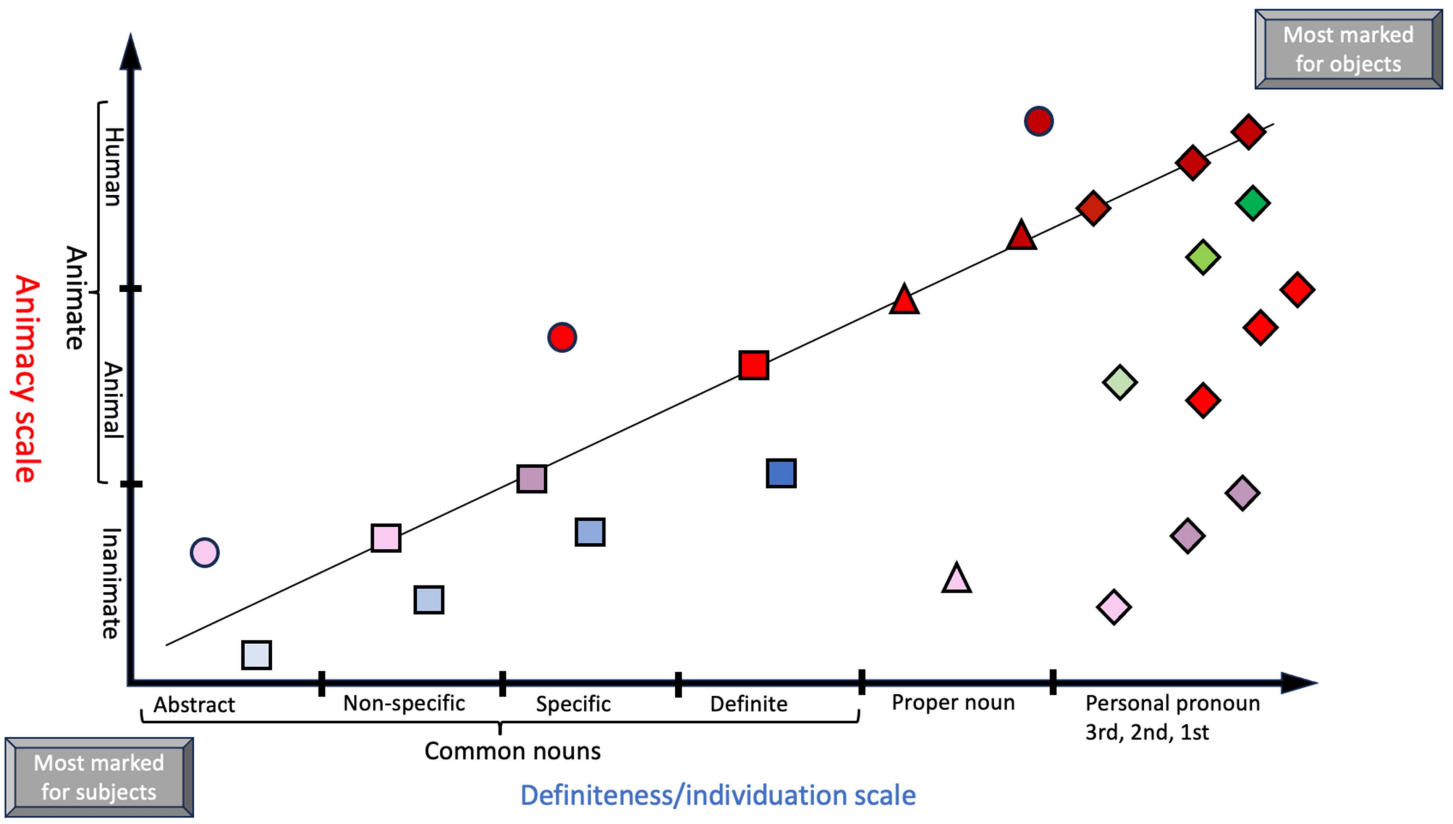
A diagram showing relative markedness of subject and object on the dimensions of animacy and definiteness, separately and combined. The placement of symbol on the plot line signifies the assumed likelihood that DOM will occur, given that the two dimensions mutually strengthens DOM likelihood (Aissen, 2003). (Note that the positioning of symbols is approximate and do not reflect data analysis). Key to symbols: Circle = animacy; square = common noun; triangle = proper noun; diamond = personal pronoun. Color key: Dark red = human; crimson red = animal; pink = inanimate; dark blue = definite noun; lighter blue = specific noun; even lighter blue = non-specific/indefinite noun; lightest blue = abstract noun; dark green = 1^st^ person pronoun; lighter green = 2^nd^ person pronoun; lightest green = 3^rd^ person pronoun. Combinations of color and shape symbol shows two-dimensional DOM, i.e., the value is determined by both animacy and definiteness scales (e.g., crimson red square = animal common noun).

Languages across the world encode verbal arguments differently, either by grouping the subject of intransitive verbs (S) and the agent of transitive verbs (A) together, with nominative case against the object (O), marked with accusative case, resulting in the pattern S = A ≠ O. Ergative-absolutive languages instead encode S and O in the same way, with absolutive case against A, which is ergative, yielding the pattern is S = O ≠ A. In a large number of languages, these systems intersect in the so-called split case systems. A widely accepted explanation emerges from the observation that, although A and O in transitive events in principle can be both definite and animate, in actual discourse, A tends to be animate and definite, and O to be inanimate and indefinite (Comrie, 1989, p. 128). In other words, transitive action typically runs from a definite and animate A towards an indefinite, inanimate O. This pattern likely reflects frequencies of how human interactions typically play out outside of language (Comrie, 1986, p. 104), or mirrors what humans think is relevant to report (Payne, 1997, p. 151). Markedness is inversely correlated with prototypes, so that any deviation from this pattern of “natural kind of transitive construction” leads to a more marked construction (Comrie, 1989, p. 128). Skewed frequency of preferred referents for subjects and direct objects (DO) of “who does something to whom or what”, creates a cross-linguistic pattern of *unmarkedness*, where it is unmarked (typical) for participants higher up in a PH to inhabit the role of subjects, but marked for participants lower in the PH, as illustrated in Figure 1.

Here, “Prototypical transitivity” contrasts with accounts of transitivity that focus on grammatical features that advance efficient transfer of action (“kinesis”) from A to O, where instead O needs to be highly individuated, defined as animate and/or definite (Hopper and Thompson, 1980, p. 256). Given Greenberg’s (1966) textual and cross-linguistic frequency criteria for protypes/markedness (Croft, 1990, pp. 71–72, 84–89, 92), Comrie’s definition of prototypical transitivity is in fact more in line with general prototype/markedness criteria. In this context, an indefinite and/or inanimate A, as well as a definite and/or animate O, would bring about more marked constructions. The former case is referred to as differential subject marking (DSM) (de Hoop and de Swart, 2008), and the latter is known as differential object marking (DOM) (Bossong, 1991; Aissen, 2003). These alignment shifts imply that some languages known as *split case languages* may shift between a nominative-accusative pattern and an ergative-absolutive pattern, when dealing with untypical subjects and objects to make explicit their syntactic functions. Nominative-accusative systems often do not overtly case-mark the subject, since agency is in focus, whereas ergative systems, being oriented towards the patient role, leave absolutive participants zero-marked, motivated by the fact that intransitive subjects are often a bit patient-like (e.g., *melt*). Languages may still differentially case mark agent-like intransitive subjects that are more volitional and in control (e.g., *jump*). It is this alternative systemic focus on agents vs. patients, respectively, that makes each system apt for split case marking at opposite ends of the action chain. Note that languages that always mark their direct objects with a certain case (e.g., accusative case), or never mark them, do not fall under the scope of DOM (Aissen, 2003), and are not in need of an explanation of how PH works.

Other syntactic constructions affected by PH include direct/inverse morphology and voice (active vs. passive) (Aissen, 1999). The primary function of passive voice is to shift the focus away from the agent performing the action to the patient receiving it by raising the patient/object in the corresponding active sentence to subject position, e.g., to maintain cohesion in discourse by focusing on a consistent topic. Direct/inverse morphological systems mark the relative position of arguments on a prominence hierarchy. Direct morphology applies when the subject referent outranks the object on the hierarchy, whereas inverse morphology applies if the speaker wishes to change this ranking order. This operation can be expressed by affixes on clausal participants and/or verbs telling who is “proximal” and who is “obviative” among two 3^rd^-person participants. The referent considered less important within the construction or in discourse is marked obviative, e.g., the possessed (compared to the possessor), or the inanimate (compared to the animate). As the marking depends on hierarchical status rather than just grammatical role, these systems do not fit neatly into traditional alignment categories like nominative-accusative or ergative-absolutive. Direct/inverse systems are relatively rare cross-linguistically, and some linguists have proposed to subsume some types of direct/inverse systems under related phenomena like topic fronting, e.g., Jacques and Antonov (2014).

### Gradience

When assigning case to a clausal participant, split case languages consider not just syntactic functions like subject and object, but also the inherent properties of the referent. These properties are a combination of linguistic and extralinguistic factors. The linguistic features include definiteness/specificity and whether the referent is rendered as a noun or pronoun; the extralinguistic aspects consider if the referent is human, animate, or inanimate, or in some languages, an abstract concept. These properties form clusters of features occupying opposite ends of gradient scales, as proposed in Croft (2003, p. 127) in the Extended Animacy Hierarchy, which really consisted of three hierarchies conflated into one, see 1a-c). A definiteness scale is proposed by Aissen (2003, p. 444) and Croft (1988, pp. 163–164), see 1d).

1) a) Person: 1^st^, 2^nd^ > 3^rd^
   b) Referentiality: pronoun > proper name > common name
   c) Animacy proper: human > animal > inanimate
   d) Definiteness scale: personal pronoun > proper noun > definite NP > indefinite NP

**Figure 2:**
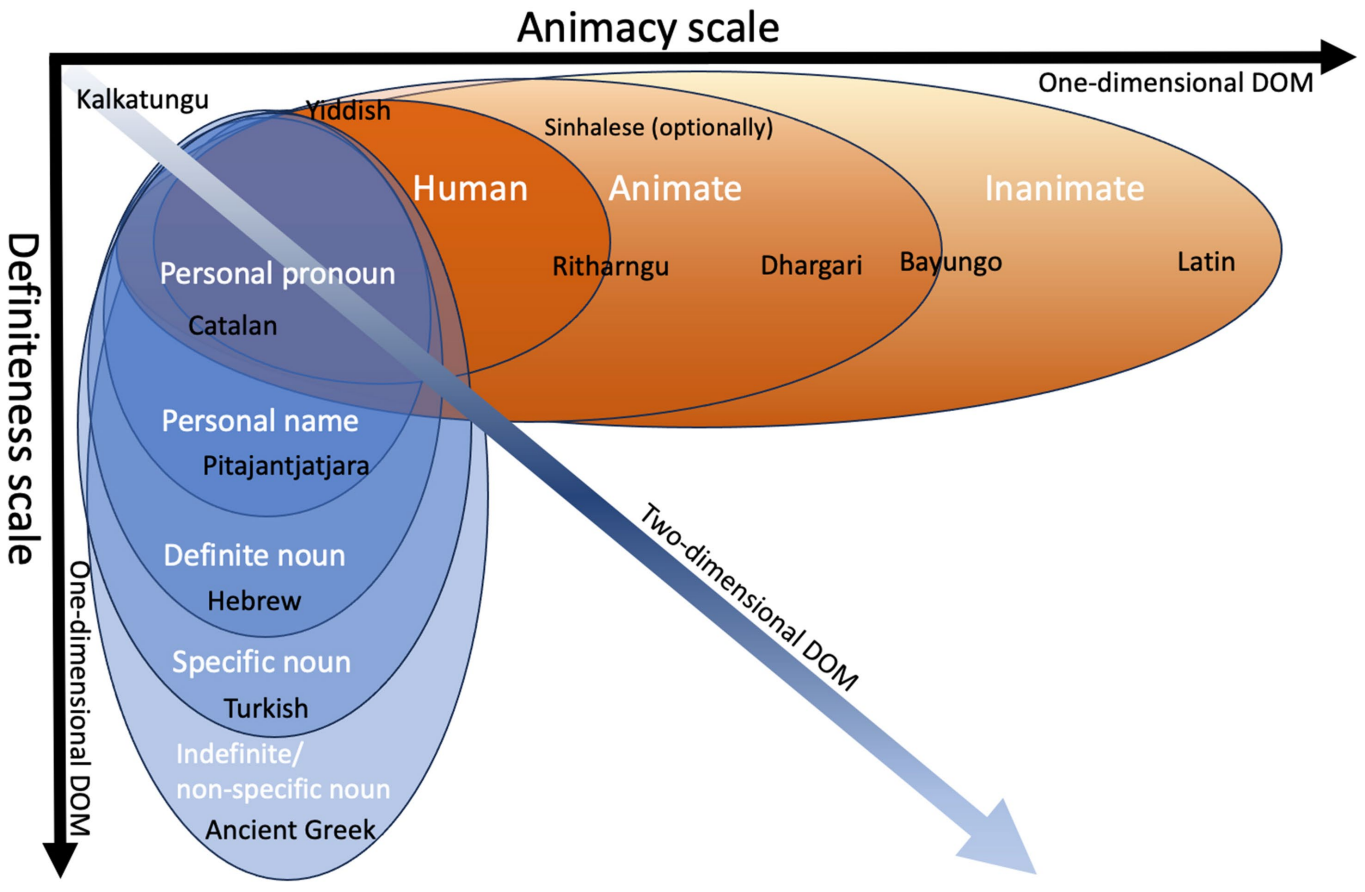
Cut-off points for a set of languages responding to DOM phenomena along one of the dimensions, animacy and definiteness. Kalkatungu never case-marks direct objects for either dimension; Yiddish case-marks some human objects only; Singhalese case-marks all animate objects, but only optionally; Ritharngu marks all human and some animate objects; Dhargari marks all animate objects; Bayungo marks all animate and some inanimate objects; Latin case-marks all direct objects. Along the definiteness scale, Catalan case-marks only personal pronoun objects; Pitajantjatjara marks only pronoun and proper name objects; Hebrew marks only pronoun, proper noun (personal name) objects; Turkish case-marks all objects except non-specific nouns (see Aissen, 2003), and finally, Ancient Greek case-marks all direct objects (but with a different case for non-specific nouns; Mardale and Karatsareas, 2020).

Generally, individuals towards the high (i.e., leftmost) end of prominence hierarchies tend to be definite while participants towards the low end of the prominence hierarchy are indefinite, with those at the far left always being definite (Dixon, 1994, p. 91). Further, a referent may be identified, but not explicitly specified, in which case they are specific, but not definite. A clustering of definiteness appears with the higher values of animacy (Croft, 1990, p. 127): A human is more often definite than an animal, and animate individuals are more often definite than inanimate objects. Pronouns likewise rank high since they per definition have definite reference (Dixon, 1994, p. 91). Nouns, by contrast, may be either definite or indefinite, and animate or inanimate, and therefore appear lower in the hierarchy. Thus, a challenging aspect with the hierarchy underlying split case languages is the lack of a clear, unambiguous boundary along these scales and instead the presence of a continuous spectrum of meanings. This also presents the second challenge, *viz.* that PH are determined by multivariate feature clusters, accounting for tendencies rather than absolute behaviors. Furthermore, PH may respond to a single parameter (one-dimensional DOM), or several parameters combined (two-, or multidimensional DOM), see Figure 2.

**Figure 3:**
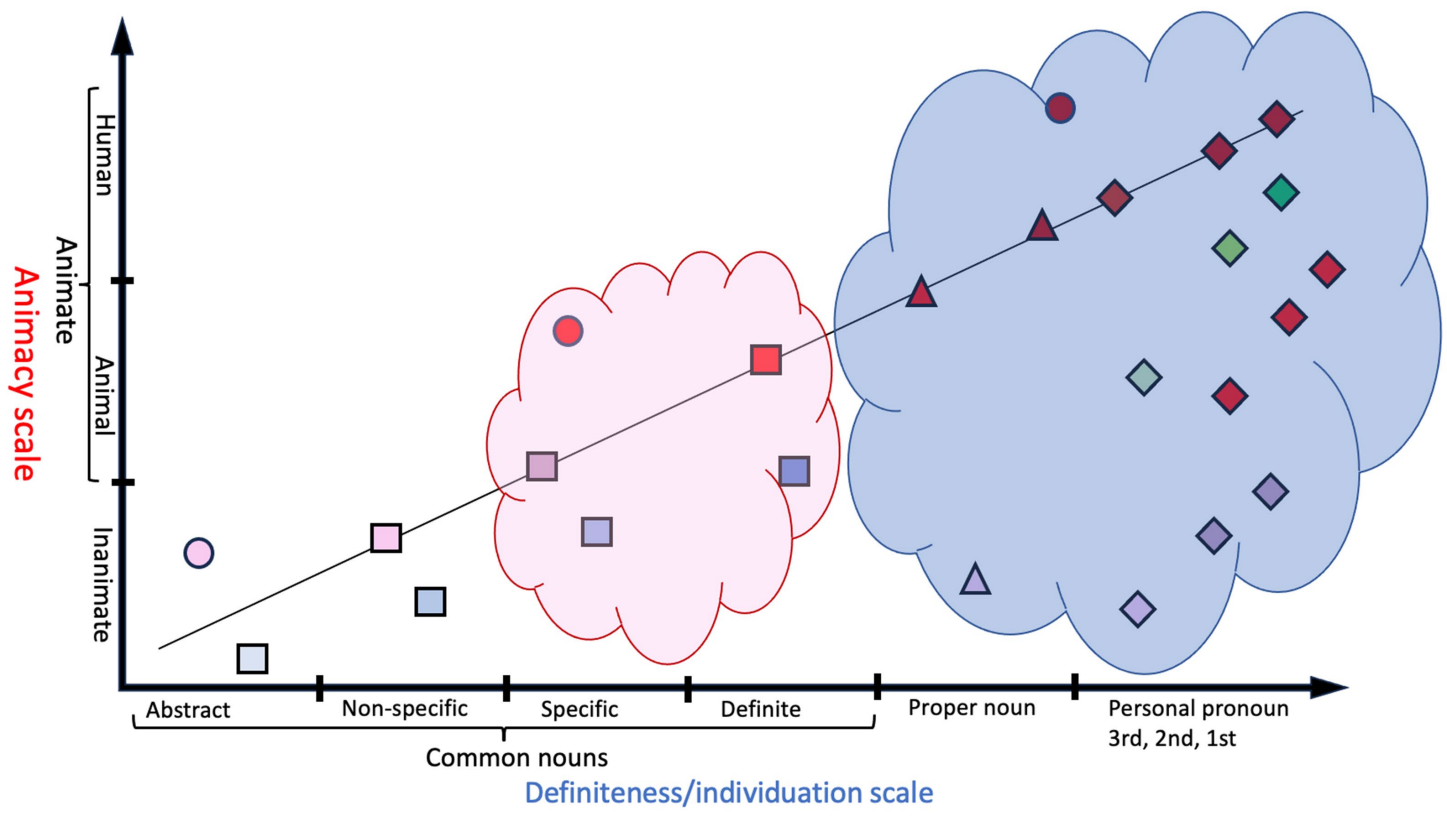
An example of DOM in Romanian of how cut-off points for case may be contiguously organized for obligatory (blue cloud), optional (pink cloud) and non-use (no cloud) (Farkas, 1978). Key to symbols: see Figure 1.

A second challenge is that split case languages case-mark direct objects in response to these dimensions at varying cut-off points along a fixed value scale. DOM may be optional, obligatory, or excluded, along different portions of a prominence hierarchy. For example, in Sinhalese, case-marking is optional, but only animate-referring objects may be case-marked (Gair, 1970). A second type is Hebrew, in which case-marking is obligatory, but limited to definite objects (Givon, 1978). Finally, a third type of which Romanian is an example, obligatorily marks the case for one group of objects (e.g., at the top of the hierarchy), optionally marks case for an in-between group, and bans case-marking for a third group (e.g., at the bottom of the hierarchy; Farkas, 1978). Notably, while optionality and cut-off points vary from language to language, the hierarchies are consistent in the sense that the order of categories are largely the same across languages (Aissen, 2003, pp. 436–437). Moreover, the parts of the scale where case-marking is mandatory, optional, or absent in each language, are contiguous along the scales (Figure 3).

Grammatical categories display various kinds of organization relative to how categories of species are carved out in biology. Surprisingly, extensions beyond and retractions within the human species and animal taxa in nominal classifier systems, as well as in PH, have been observed in multiple languages. Category structure can be partially culturally determined and cultural knowledge can have an impact on grammar by imposing constraints on morphosyntax (Aikhenvald, 2000, p. 319). Langacker (1994, p. 39) points out that “a specific cultural practice or belief motivates the otherwise unexpected membership of some entity in a conceptually grounded category of grammatical significance.” Instead of looking at this as mismatches relative to biological taxa, it is profitable to analyze category membership and cross-linguistic variance in borderlines in terms of what is deemed standard, common, or frequent within a culture, and in principle no different from how prototypes and markedness is defined elsewhere in language.

### Scope

The topic of prominence carves out some limitations to focus areas. First, ranking participants in prominence within a clause can only be done with a minimum of two participants. The original definition of “animacy hierarchy” was made with reference to agent, patient, and transitive propositions (Aissen, 1999, p. 674). Second, a transitive trajectory playing out in space–time requires verbs depicting temporally measurable events. This corresponds to Vendler’s (1957) verb class known as “accomplishments,” defined as dynamic and durative events with an inherent endpoint. This delimitation can be justified from the viewpoint of prominence as well, although indirectly. Split ergativity, a central facet of prominence hierarchies, correlates with verbs of action, while other types of verbs may follow a different pattern (Tasaku, 1981). In some split ergative languages, the split aligns with semantic properties of verbs, such as the degree of control or volitionally associated with the action (Bohnemeyer, 2004), which again correlates with completed actions. Thus, although the interface between prominence hierarchies and Vendler’s verb classes is not a concluded matter, these fields seem to overlap in the sense that they both intersect with the aspectual properties of verbs.

**Figure 4:**
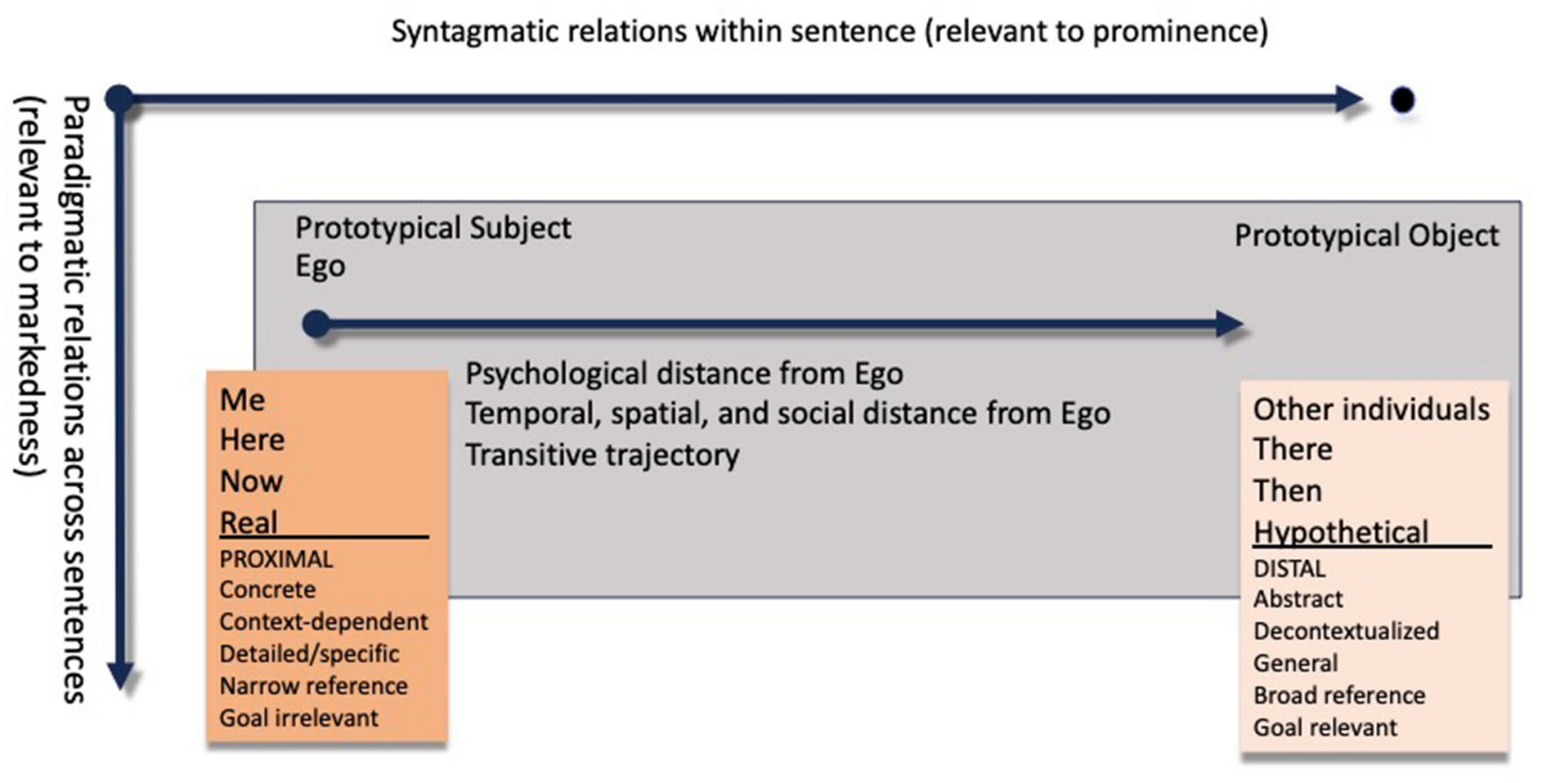
Syntagmatic vs. paradigmatic relations and prominence within a transitive sentence within CLT. Temporospatial distance from ego refers to a verbal action towards the direct object with an inherent endpoint (accomplishment).

Third, a focus on syntagmatic relationships within sentences is warranted since this is where comparison between clausal participants plays out. A clarification of prominence and markedness with reference to syntagmatic vs. paradigmatic relations might be helpful. The values within a grammatical category that stands in a markedness relation must be logically independent of each other. However, this is not the only requirement and in order to qualify as a markedness pattern, the values in question must be paradigmatic alternatives (Croft, 1990, p. 69). In other words, markedness is restricted to “a relation between features which are mutually exclusive” (Greenberg, 1966, p. 57). For example, an animate direct object stands in a paradigmatic relationship to an inanimate DO, since one and the same sentence cannot have both an animate and an inanimate DO at the same time; they must appear in different sentences. Among the two, the animate is the unusual one and receives overt case marking, e.g., accusative case, the inanimate is unmarked. While being paradigmatic alternatives, these values must also exist at a higher level of abstraction in grammar, in this case the category of “direct object.” This means that the markedness and prominence dimensions are orthogonal to each other, but also that paradigmatic opposition is irrelevant to the relative prominence between verbal arguments within a sentence (see Figure 4).

Our analysis will make use of the concept “psychological distance” to explain the above-mentioned challenges, in which temporal distance is one dimension affecting levels of construal. Here, we make explicit that exchanging the present tense with either past or future tense (i.e., more temporally distant from Ego) does not have an effect on the level of construal for the clausal participants. This is because the temporal dimension is measured along the transitive trajectory, as syntagmatic relations, and not along the paradigmatic axis where the various tenses alternate between sentences. This is logical since it is within a particular sentence that participants compete for prominence. Evidently, the axis along which prominence is measured and the temporal dimension *need to converge*, otherwise these two dimensions would not merge as the same psychological distance.

### Preliminary conclusion

Despite prominence hierarchies are ubiquitous in language, it has been notoriously difficult to understand what exactly they are about. It has been challenging to find a common denominator for the whole of the “Animacy hierarchy”, as Helmbrecht et al. (2018) point out, and it is not surprising that this hierarchy has been interpreted in different ways in the literature. In fact, a variety of names has been assigned to it since it was first discovered: “Lexical hierarchy” (Silverstein, 1976); “Nominal hierarchy” (Dixon, 1979, 1994, p. 85); “Animacy hierarchy” (Comrie, 1989); “Empathy hierarchy” (Kuno and Kaburaki, 1977; DeLancey, 1981); “Hierarchy of reference” (Zwicky, 1977); and “Prominence hierarchy” (Aissen, 1999; Lockwood and Macaulay, 2012). We therefore propose to cast a broader net by looking at these phenomena within cognitive psychology, specifically within the framework of the Construal Level Theory.

## Construal level theory

In psychology, it has been common to model a variety of mental representations as taking place within a mental or virtual “space” (Shepard, 1962). From the perceptual representation of colors (Krantz, 1975; Smith and Pokorny, 1996; Logvinenko, 2015) to those of human faces (Valentine et al., 2016; Leopold et al., 2001) or of emotional states (Russell, 1980, 2003; Posner et al., 2005), the stimuli corresponding to values or “points” within a multidimensional space, often defined by only three orthogonal polarities (as for color space) or even just two (as for the emotional circumplex). In some of these models, there can be a central point that corresponds to an average or neutral point, where values along the dimensions cancel each other out (as the central achromatic grey in color space; or the sexless, unemotional, and ethnically-hybrid prototypical face). Distance between positions in such spaces indicates how features or entities differ or the degree in which they are proximal/distal from each other or whether they are opposites (e.g., as with opponent colors).

However, recent modelling within psychology, where social dimensions are fundamental, posit psychological spaces where the central, or zero, locations are always in relation to self and one’s current spacetime coordinates; that is, Ego and the “here-and-now”. Increasingly dissimilar features from the Ego or the moment’s spacetime coordinates are construed as increasingly mentally distant events or things, as well as increasingly abstracted away from the present and egocentric viewpoint. One such account is known as the “Construal Level Theory” (Trope et al., 2007; Trope and Liberman, 2010, 2012), which would seem most relevant from a linguistic perspective. This account derived from the simple observation that “distance” and level of abstraction appear to be related in people’s minds. That is, the model makes explicit that, while distance can happen in different domains (spatial, temporal, social) as well as in terms of hypotheticality, all these dimensions are associated and affect one another, thus subsumed under the unified concept of *psychological distance*. Such a mental distance also implies the subjective experience or feeling that something is close or far away, similar, or dissimilar, in time, space, and social distance from self.

Specifically, the central or zero psychological distance corresponds to the most concrete and most similar situation or object to a Self or Ego situated in the *specious present*, the time duration wherein one’s perceptions are considered to be in the present (Kelly, 1882; James, 1893). As thoughts or perceptions are further removed from the Self and the Here-and-Now, or from material reality, mental construals become increasingly distant and, importantly, increasingly abstract. They zoom out from the specific, detailed, subordinate, contextual aspects of a situation or class of objects and extract only the essential, often goal-relevant, features. Thus, mental construal seems a useful cognitive tool that allows human minds to mentally maneuver events and objects in past or future contexts, rather than only living in the present, and to refer to hypothetical and counterfactual events rather than the confines of the real and material situation. Traversing along psychological distance enables people to cross the distances that separate the Self from the Others, the Now from the Then, and Here from There.

Construing psychologically distant or distal events allows the capacity for “mental travel” (Soderberg et al., 2015) and, together with the process of abstraction from a single, concrete, event, may constitute the key mechanism for going beyond the immediate or proximal experience, like when reminiscing, speculating, and making predictions. Importantly, according to CLT (Liberman and Trope, 2008), psychological distance influences the way we represent the world, how we categorize it and communicate about it with others. With respect to social relationships, given that psychological distance is defined in CLT as the extent of divergence from the direct experience of me (Liberman and Trope, 2014), this results in a hierarchical scaling of Self versus proximal and then increasingly distal others, along socially perceived similarity/dissimilarity with other individuals (e.g., in culture, attitudes, appearance), familiarity versus unfamiliarity, as well as a hierarchical scaling of the others in relation to the ingroup (e.g., kin, clan) versus the outgroup classes. According to CLT, the mind understands psychological distance abstractly and literally, in social as well as in physical terms, operating along continuous scales, often without a fixed maximal point of distance. As psychological distance increases away from the proximal to the distal, peoples’ representations of objects and events become ever more abstract.

CLT was forged from the results of an impressive number of empirical studies. For example, researchers found not only that proximal concepts triggered concreteness and individuation, but also that this individuation is linked to the first-person perspective. By using an implicit association test, first person pronouns like “ours, ourselves, at our place, for us,” and “we” were associated with words that denoted exemplars (*beet, poodle, belt*), while third person pronouns “they, theirs, for them, at their place” were instead associated with categories (*vegetables, animals*, and *clothes*) (Bar-Anan et al., 2006, experiment 3B). Subjected to the same kind of test, participants non-consciously associated socially proximal concepts like “friends, parents, buddies,” and “siblings” with concrete words, on the one hand, and socially distal concepts like “enemies,” “strangers,” “opponents,” and “anonymous person” with abstract concepts, on the other (Bar-Anan et al., 2006). Thus, there seems to be a bidirectional, interdependent relationship between level of construal and social distance. Participants who explained individuals’ behavior using global dispositional qualities also tended to perceive them as more socially distant, compared to participants who explained the very same behavior in terms of concrete, situational factors (Stephan, 2006: see Bar-Anan et al., 2006).

Pointing to the origin of the concreteness factor, proximal level construals are context-dependent while distal construals are decontextualized. In one study, people’s construal of distant-future activities stated the goals of the activities, whereas the construal of near-future activities stated the means and/or the spacetime coordinated for achieving these goals (Liberman and Trope, 1998). The reality people experience when immersed in a specific context are typically more detailed, as they are tied up with practicalities and the “how” aspects of the activities. These subordinate-level construals preserve a stimulus in minute detail, while emphasizing its unique features rather than focusing on a situation’s similarity to other stimuli. By contrast, decisions based on distal level construals along the temporal dimension are supervised by desirability concerns—the why’s—while downplaying feasibility, potential contextual constraints, and the means necessary for enactment. Because a distant object is decontextualized, only the gist of available information, the superordinate time-stable core features of the event, are mentally represented and considered, which often results in planning fallacies (Trope and Liberman, 2012).

In this research, participants use broader categories in distal than proximal level construals across dimensions. In the social dimension, people tend to describe outgroups using more abstract qualities compared to ingroups, (Fiedler et al., 1993; Werkman et al., 1999), and their properties as more structured and predictable (Linville et al., 1996). Outgroups are perceived as less differentiated into subgroups (Brewer and Lui, 1984; Linville, 1982; Park et al., 1992), and also as more homogenous than ingroups (Jones et al., 1981; Park and Judd, 1990; Park and Rothbart, 1982). As in the social dimension, participants tested in the temporal dimension used broader categories to classify objects for distant-future than for near-future situations, which were instead organized in narrower categories of concrete objects more unstructured and incidental (Liberman et al., 2002, Study 1). Likewise, in the probability dimension, improbable events, being removed from direct experience, seem more distal and instigate participants to categorize objects in fewer, broader groups than those imagining probable events (Trope et al., 2007).

Fewer dimensions underlie people’s judgment of temporally distal than proximal events. In an event-rating task, distant-future preferences could be statistically accounted for within two- to four-factor solutions, whereas the near-future preferences always required one factor more to account the same amount of variance (Liberman et al., 2002, Study 2). Thus, near future preferences proved to be more complex, harder to reduce to general underlying dimensions, and were determined by a larger set of distinct factors than the corresponding distant-future preferences, which represented preferences in simpler structures.

In line with this simplification, distant-future events seem to represent more prototypical cases than near-future events. Participants were asked to write down and valence the events they expected to experience, during a good or bad day, in either the near future (tomorrow) or the distant future (a day a year ahead) (Liberman et al., 2002, Study 3). The near future events were described as more diverse than in the distant-future day, and prototypical and more extreme experiences were expected in the distant than in the near future. The researchers concluded that distant-future experiences were more schematic, since intracategory homogeneity was greater, and there was greater intercategory divergence (Trope and Liberman, 2003). In sum, as temporal distance increases, future events are represented more parsimoniously and with greater abstraction.

Several experiments suggest the four dimensions are cognitively interrelated and integrated. For example, abstraction increases with distance and decreases with proximity when the temporal and social dimension are conjoined. Personality ratings were more similar across social roles when participants were thinking of themselves on a day a year later than on the following day. The distant self was thought of in a more integrated, structured manner, and the near self was more contextualized and fluid (Donahue et al., 1993). Another study (Nussbaum et al., 2003) exploited the tendency people have to identify behaviors from underlying, dispositional traits (attribution theory; Trope and Burnstein, 1975). With increased temporal distance, participants were more likely to attribute behavior to personality traits rather than to situational demands. Reasoning that traits are tokens of generalized representations, they found that more abstract, higher-level, construals were used to predict and explain distant-future behaviors. Moreover, the finding that first-person pronouns were associated with exemplars and third-person pronouns with categories translated to the temporal dimension as well, since the same exemplar/category pronominal stimuli were used in an experiment on associations with “near time” (*a second, a minute, now, immediately, soon*) vs. “distant time” (*a year, a decade, later, last year, long ago*) (Bar-Anan et al., 2006, experiment 1B).

Thus, whether investigated separately or in combination, the dimensions produce similar results and participants organize items in broader and more general categories when perceived or imagined at greater distances compared to in proximal level construals. Table 1 summarizes CLT results and predictions.

**Table 1:** Construal level characteristics at proximal vs. distal distances of events and objects (Soderberg et al., 2015; Trope and Liberman, 2003).

Proximal-level construals	Distal-level construals
Concrete	Abstract
Complex	Simple
Exemplars or narrow categories	Broad categories
Individualized information	Dispositional information
Details	Gestalts
Subordinate	Superordinate
Secondary features	Primary features
Specific behaviors	Broad traits
Situational information	Aggregate information
Situation-specific demands	Goal relevant
Contextualized	Decontextualized
Feasibility concerns	Desirability concerns

Hence, we hypothesize that prominence phenomena (hierarchies and categories) fall into the same kind of grid as here described within CLT. Next, we will look at some of the challenges that former analyses have created in presenting a unified understanding of these phenomena.

### Unsolved conundrums of prominence hierarchies and category structure

In this section we organize previously researched aspects of prominence into five overarching topics: A. vantage point and animacy, B. individuation and narrow reference phenomena, C. fronting mechanisms, D. abstraction, and E. linguistic aspects of cultural variance and flexibility. Each of these umbrellas subsume a variety of manifestations in grammars which we outline and exemplify under each point. Notably, several conundrums arise because previous understandings of PH and linguistic categorization are conceptually incoherent on a superordinate level or explain only a portion of PH behavior.

### Vantage point and animacy

#### The first conundrum: why are speech-act participants exclusive in prominence hierarchies?

A prominence hierarchy is modified by a person > non-person contrast along the gradient 1^st^ > 2^nd^ > 3^rd^ person pronouns (“I” > “you” > “he/she/it” in the singular). Person hierarchies manifest themselves most famously in direction-marking systems, which mark transitive actions that comply with the prominence hierarchy (i.e., the agent never ranks higher than the patient), with a direct verbal affix, and actions that contradict the prominence hierarchy as “inverse” (Croft, 1990, pp. 136–137). These markers are neutral with regard to the syntactic roles S, A, and O (the addition of inverse affixes do not change the syntactic functions relative to the direct construction), and do not appear on intransitive verbs (Jacques and Antonov, 2014). Direction marking languages leave the direct configuration unmarked, or mark both, as in the Tibeto-Burman language Jyarong, where the direct suffix *-a* occurs with speech-act participants (1^st^ and 2^nd^ person, or SAP) acting on 3^rd^ person participants (SAP →3^rd^), and the inverse *-uk* suffix occurs with 3^rd^ → SAP.

The most common pattern cross-linguistically distinguishes SAP from all other noun phrases, including 3^rd^ person pronouns (DeLancey, 1981, p. 628). Number, DOM, and direct/inverse systems all give priority to the 1^st^ and 2^nd^ person pronouns, or the individuals engaged in communication, one as the speaker and the other as the listener. In no known language are 3^rd^ person pronouns grammatically ranked over SAP. Languages generally rank 1^st^ and 2^nd^ equally or rank 1^st^ > 2^nd^ (but the reverse order, 2^nd^ > 1^st^, exists in Algonquian languages). Still, a noteworthy universal constraint applies to the internal ordering of SAP: the single option not attested in any language so far is inverse marked 1^st^ → 2^nd^ while 2^nd^ → 1^st^ is marked as direct, which warrants primacy to 1^st^ person pronouns over 2^nd^ (Jacques and Antonov, 2014).

As examples of joint vs. gradient SAP ranking types, consider these two Tibeto-Burman languages. In Kham grammar, agents are conceived of as unmarked (2a-c), but 3^rd^ person is not and needs to be marked for agency with ergative case (2d). Participants raised from a lower PH-position to a role reserved for the higher-ranking SAP, need special morphological marking to be accredited this status. Conversely, 1^st^ and 2^nd^ person need to be agreement marked as DO in object position (2a, 2b), but a 3^rd^ person DO does not (2c).

2) a) nga:      nǝn-lay         nga-poh-ni-ke.
        I          you.OBJ      1^st^.Ag-hit-2^nd^.Pat-PERF
      ‘I hit you.’
    b) nǝn       nga-lay          nǝ-poh-na-ke.
        You      1^st^.OBJ          2^nd^.Ag-hit-1^st^.Pat-PERF
       ‘You hit me.’
    c) nǝn       no-lay            nǝ-poh-ke.
        You      he.OBJ            2^nd^.Ag-hit-PERF
       ‘You hit him.’
    d) no-e     nǝn-lay          poh-na-ke-o.
        He-**ERG**    you.OBJ     hit-**2**^**nd**^**.Pat**-PERF**-3**^**rd**^**.Ag**
       ‘He hit you.’

In contrast, for the Nocte language, the hierarchy 1^st^ > 2^nd^ > 3^rd^ person is predicted. Agreement is marked on the verb, always prioritizing SAP over 3^rd^ person: agreement with 3^rd^ person occurs only when no SAP are present. The two SAP are nevertheless internally ranked with the PH headed by 1^st^ person, since both 2^nd^ → 1^st^ (3b), and 3^rd^ → 1^st^ (3c) requires the inverse suffix *-h*:

3)  a)  nga-ma      nang        hetho-e
          I-ERG        you         teach-1PL
        ‘I will teach you.’
     b) nang-ma      nga        hetho-**h**-ang
         You-ERG        I         teach-**INV**-1^st^
       ‘You will teach me.’
     c) Nga-ma      ate            hetho-ang
         I-ERG       he              teach-1^st^
        ‘I will teach him.’
     d) Ate-ma     nga-nang       hetho-**h**-ang
         He-ERG    I-ACC          teach-**INV**-1^st^
       ‘He will teach me.’

Since DeLancey (1981, p. 644), direct/inverse systems have often been described within an Empathy hierarchy, arguing that discourse participants represent attentional “natural starting points,” while also motivated by the proximal/obviative distinction in 3^rd^ person in these languages (Jacques and Antonov, 2014, p. 304), see 4):

4) SAP > 3^rd^ person pronoun > human > animate > inanimate

A typical conversation between single individuals in face-to-face interaction takes place with some asymmetry towards the 1^st^ person as the initiator: the speaker has the primary role of delivering information and guiding the conversation, and decides what to share, controlling the flow of information. This perspective is qualitatively different from other perspectives (Tressoldi et al., 2017).

The primacy of 1^st^ person pronouns in PH also finds a parallel in CLT. Across a wide array of experiments, participants used their own vantage point as basis for conceptualizations about events, objects, and actions with abstractions occurring in increasing psychological distances from Self (Trope and Liberman, 2010). Psychological distance can imply temporal (Trope and Liberman, 2003; Wakslak et al., 2008), or spatial distance (Henderson et al., 2006), unlikeliness of occurrence (Wakslak et al., 2006), and distance along the social dimension, either in terms of an actor being dissimilar or emotionally distant to the perceiver (Liviatan et al., 2008). All of these dimensions matter in the context of SAP.

How a conversation plays out is acutely context-dependent, hinging on the interlocutor’s responses and cues in the physical environment (Dohen et al., 2010). The event is factual and dynamic in the sense that it manifests itself in real time with turntaking participants. Experimental evidence suggests that as one moves away from MHN, this changes. Along the temporal dimension, perceivers put less weight on situation-specific states when predicting others’ behaviors in near future than distant future events (Nussbaum et al., 2003). The use of social media dissociates interconnectedness of time, space, addressee specificity, and context-dependency in communication. Joshi et al. (2016) found that addressing psychologically distant versus psychologically close audiences has direct effects on communication. Taking an expansive vs. contractive relational scope of addressees in non-face-to-face dialogs alters language use towards high-level, decontextualized messages that are situationally stable and applicable across contexts.

In addition, the nexus of SAP in grammars can be explained with reference to experiments on face-to-face conversations. The listener’s role in a conversation goes beyond that of a “hearer”; it demands a collaborator’s role who is “a full partner in creating the dialogue,” by the use of facial displays, collaborative, interactive gaze patterns, gestures, and brief vocalizations, even when not taking up the speaking turn (Bavelas and Gerwing, 2011). Face-to-face speech communication is a multimodal process and involves not only linguistic but also psychological, affective, and social interaction (Dohen et al., 2010). Speech-act participants relate to each other as individuals, mind to mind. The interlocutors engage mentally in physical acts as well as in mental and emotional interplay, read each other’s facial cues and actively strive to understand the other person’s intentions and thoughts through “grounding” processes (Bavelas and Gerwing, 2011). Conversation is a two-way process: distracted addressees not able to collaborate in conversation impair speakers’ storytelling (Bavelas and Gerwing, 2011). Overhearers differ from addressees by not partaking collaboratively and they do not contribute to mutual understandings. These experimental data support the cross-linguistic roles of SAP in PH.

Social distance affects first-person vs. third person perspective taking (Trope and Liberman, 2010). Research shows that third-party viewpoints are less context-sensitive than first-person viewpoints: Personal memories of behaviors recalled from a third-person perspective produced dispositional descriptions rather than situational terms, whereas the opposite was true for first-person perspectives (Frank and Gilovich, 1989; Nigro and Neisser, 1983). Research within perspective-dependent recall also revealed that perceivers tend to remember more global, dispositional qualities in recalling events from a third person perspective than from a first-person perspective. Imagining performing an activity from a first-person perspective were more vividly depicted than when participants imagined the same activity from a third-person perspective, which instead brought about more abstract and less detailed reports of activities (Libby and Eibach, 2002, Study 4). In terms of CLT, this means that a third-person perspective imposes more psychological distance and higher-level construals than first-person perspective (Trope and Liberman, 2010, p. 448).

In sum, the existing research supports the idea that distance from 1^st^ person towards 2^nd^ and 3^rd^ person pronouns in PH converges with psychological distance emanating from first-person perspective in CLT, and empirical research on face-to-face communication pairs up with the SAP nexus found in many grammars.

#### The second conundrum: why does linguistic animacy differ from biological animacy?

In PH, even finer-grained scales rank animals lower than humans, although human animacy is no different from that of animals, biologically speaking. Despite this conspicuous mismatch, it is still more common among linguists to use the term “the animacy hierarchy” than any other term (e.g., Gardelle et al., 2024; Haude, 2024), tacitly accepting a notion of “linguistic animacy” but not stating what that is. Yamamoto (2006, p. 31) argues that “the General Animacy Scale” is based on a kind of hierarchy of animacy *per se* with the assumed natural taxonomy regarding the hierarchy of “living things,” but also that “hierarchical classification of animate beings and inanimate objects is the product of our subjective view of these entities.”

Following this lead, we propose to integrate a linguistic theory of prominence into a wider theory of cognitive construal to offer an alternative, multi-disciplinary view. Recently, a person-centric bias in cognitive representation was robustly documented as the primary way people understand the external world (Kalkstein et al., 2020). Participants were tested for their tendencies towards perceptual mappings of “object-level identities” or towards “relational mappings,” after they observed pairs of identical scenes with minimal manipulations. It was found that the presence (vs. absence) of a person in a scene lead participants to engage in relational mappings, suggesting that the distinct qualities of objects are secondary to how those objects relate to and interact with the person in the scene. Moreover, they were more likely to construe animals in terms of their relationship to humans than to construe humans with reference to their relationship to animals, suggesting person-centric cognition. Hence, people constitute cognitive anchors as to how a scene should be mentally represented and the meaning of objects as well as animals within a scene, becomes defined by their relationship to that central person, therefore giving primacy to people over animals and inanimate objects (Kalkstein et al., 2020, p. 2).

#### The third conundrum: why are direct objects more general, non-specific, and abstract than subjects?

Discussions on prominence hierarchies constantly revolve around the clustering of agency and animacy, definiteness/specificity, and the topicality of clausal participants towards the high end of PH (Croft, 1990, p. 127). This clustering has been neither explained, nor have the causes for the absence of these features in direct objects been sufficiently contemplated. Agency is an interactive notion rooted in social landscapes involving “relational reasoning” that classifies them by the role they occupy in relationship to other objects within an event or scene instead of categorizing entities by their perceptual features (Gentner, 1983; Goldwater and Markman, 2011; Kalkstein et al., 2020). The agent and patient roles in canonical active sentences are well known but their distinct asymmetry has not been explicitly addressed as a potential underlying cause of PH or the protypicality of subjects and objects in transitive sentences.

The notion of agency finds a parallel in CLT-research, where it has been shown that powerful agency generates greater distance to and greater abstraction of target categories. An experiment (Smith and Trope, 2006) found that elevated power increases the psychological distance one feels from others, demonstrating a direct relationship between power activation and abstraction. Participants first completed a writing task that activated the experience of either high or low power; subsequently they completed a categorization task to measure inclusiveness of atypical exemplars. Results showed that high-power primed participants were more inclusive in their categorization than low-powered primed participants, demonstrating that power priming leads to more abstract thinking and thus greater breadth of categorization (Trope et al., 2007). The exact same tendency we see in prototypical transitivity: agents are measured by their potency and correlates with broader and less specific direct objects. Thus, in our present proposal, the distance between agent and patient is represented in terms of a mental timeline from the agent, simultaneously representing temporal (in terms of the time it takes to perform the action), the social distance felt by at powerful agent, and a spatial distance between the agent and the patient prior to action. The remaining clustering features relate to individuation and topicality, to which we turn in the next couple of sections.

### Individuation and narrow reference

Languages have a number of techniques to single out exemplars or limited sets rather than generic groups or categories. One way is to let the addressee know that one has a specific item in mind without disclosing which one (specificity), another technique shares this knowledge with the addressee or assumes it to be common knowledge (definiteness). Another way actively points out the referenced item among a set of potential items (demonstratives), some add a unique identifier to contestant candidates (epithets), and some are themselves unique identifiers (names). Finally, some use situated reference points as indirect deixis for identification (kinship terms). These devices have in common that they seek to draw attention to an exemplar, the ultimate narrow reference, rather than a group of items, and common nouns typically refer to classes of items without inherently referencing single items. Nevertheless, some of these common nouns are considered more “animate” than others and are conceived of as individualized, when occurring in a group of entities (generic plural) or as a narrow set of entities (dual, trial, and paucal plurals). In contrast, for the remaining nouns, distinguishing similar entities is irrelevant (unspecified for plurality). These individualized and narrow referenced items all appear towards the higher end of PH, and we believe this is in demand of an explanation that goes beyond animacy.

#### The fourth conundrum: why is individuation more prevalent in higher than lower ranked PH categories?

Individuation is more pertinent with participants higher up in the PH than those in lower rank. Number distinction is one way of expressing individuation. To specify plurality is marked relative to specifying singular status by an additional morpheme (Croft, 1990, p. 89), but often required in high-ranking nominals. Thus, for some nouns within certain languages, the opposition between singular, plural, and sometimes dual, is significant, while for other nouns, it is considered irrelevant. This phenomenon is known as “split plurality” and affects “any of the mechanisms used to mark plurality,” including agreement between verb-argument or noun-modifier, direct marking of a noun or noun phrase (Smith-Stark, 1974). Pronouns and nouns referring to animate individuals, including humans, may have a number distinction that is not found with common nouns that designate inanimate things. For example, Tiwi grammar distinguishes between singular and plural of humans, e.g., *wuɹalaka* “young girl” and *wuɹalakawi* “young girls”, but not of ants, e.g., *waliwalini* “ant/ants” (Osborne, 1974, p. 52), and in Kharia, separate forms represent cats in the singular and plural, *biloi* “one cat” and *biloiki* “cats”, but only one form represent stone/stones: *soreŋ* (Biligiri, 1965, p. 36).

When languages have the possibility of inflecting for number, they display the same kind of graded differences in cut-off points as we observed with animacy and definiteness (see Figure 2). They adhere to a hierarchy where within a certain language, 1^st^ and 2^nd^ persons can be distinguished in number (e.g., be agreement marked for number on the verb), but the same grammatical process does not apply to referents lower in the hierarchy such as the 3^rd^ person. For example, in Georgian (Vogt, 1938), only the 1^st^ and 2^nd^ person pronouns are agreement-marked on the verb. Again, cut-off points are language-specific, and, in Kwakiutl, a reduplicating-distributive plural applies mandatorily only to the 1^st^ person pronouns. In 2^nd^ and 3^rd^ person, by contrast, plural is optional (Boas, 1911, p. 444). Pronoun systems may preferentially mark plurality only in SAP, so that Guaraní distinguishes for plurality and inclusive/exclusive in the 1^st^ and 2^nd^ persons, but a common form haɂé represents singular/plural 3^rd^ person referents (Gregores and Suárez, 1967, p. 141). Accordingly, Croft (1990, pp. 111–112) proposed a hierarchy for the markedness for number relations as in 5):

5) 1^st^ and 2^nd^ person pronoun > 3^rd^ person pronoun > proper names > human common noun > nonhuman animate common noun > inanimate common noun

At the same time, animate categories are left unspecified for number when they appear in object position and seen in relation to its usefulness for humans as game/food (e.g., the Norwegian sentence in 6).

6) Han    har    skutt    mye    elg.
     He    has    shot     much     elk.SG
    ‘He has shot a lot of moose.’

Holding this example against the findings of Kalkstein et al. (2020) that entities in a scene are defined against the presence of humans, it becomes evident that individuation is a feature of prominence, not of whether a clausal participant is animate or not.

In sum, categories higher up in PH tend to be individualized, while categories lower in the PH, are not.

#### The fifth conundrum: how can kinship terms, epithets, and personal names refer to humans while ranking above the human category?

Split plurality may adhere to narrower borderlines than animacy, sometimes to humanness, but often to the even narrower class of “kin.” In Kpelle, only the subgroup of nouns referencing humans that consists of kinship terms are pluralized (Welmers, 1969, p. 82). Plurality may favor close relationship terms, e.g., *brother-in-law*, *wife* in Tlingit, while inanimates optionally take collective plurals (Swanton, 1968, p. 169). Based on split plurality examples from a variety of languages, Smith-Stark proposed the hierarchy of features controlling split plurality of nouns as in 7):

7) Speaker > addressee > kin > rational > human > animate > inanimate

The positioning of kinship terms between the 2^nd^ and 3^rd^ person pronouns categories, e.g., in Gumbayŋgir, makes sense within proximal level construals. Kin are in a direct personal relationship to the ego, whereas 3^rd^ person referents need not be. In kin term split plurality languages too, the proximity of interpersonal relationships with individual attachments trigger individuation strategies, other nouns do not.

Kinship terms, titles, epithets, and names occupy intermediate sections of PH, but always rank above human nouns. Split ergativity systems may rank kin terms and proper names equally and positioned between personal pronouns and human nouns. In Gumbayŋgir, kinship terms but no other nouns must be marked with accusative case in object position while also being mandatorily marked with ergative case when in subject position (Silverstein, 1976). This behavior applies to titles and epithets as well (Silverstein, 1976: “section names”). An epithet functions as an identification device, separating an individual from similar individuals, attributing specific characteristics to a person, e.g., *Richard the Lion-Hearted*. Its function is reminiscent of definite particles in that it singles out an individual for unique identification. Whenever personal names are ranked, they constitute a class between personal names and other nouns (Helmbrecht et al., 2018), see 8):

8) 1^st^/2^nd^ person > 3^rd^ person > personal name > human common noun > animate common noun > inanimate common noun

Although cross-linguistic evidence remains scant for the existence of proper names as an independent category in this context, there seems to be an intuitive appeal to names being high in PH. In Romanian, the DOM marker *pe* required for human and specific nouns is also always used with pronouns and proper names (Onea and Mardale, 2020, p. 357). In Arabana, a tripartite split ergative/accusative case-marking system ranks personal names second: SG pronouns > personal names > common nouns. Other tripartite systems collapse personal names with adjacent PH categories: common nouns (Diyari), 3^rd^ person/human nouns with demonstratives but not common nouns (Yidin), or personal pronouns with human/animate common nouns (Manipuri, Dhankute Tamang, and Thakali; Helmbrecht et al., 2018).

While names pattern with categories towards the high end of PH, they also associate with demonstrative and personal pronouns (in other languages besides Yidin). Norwegian has two sets of grammaticalized 3^rd^ person personal pronouns with deictic and specificity semantics preceding personal names (Johannessen, 2008). One set are the psychological proximal demonstratives (PPD) labelled “preproprial articles” in Johannessen (2008, p. 170). These introduce personal names/kin terms of close family relations, e.g., *mother*, *father*. PPD agree with the semantic gender of the referent, e.g., *a/hu/ho Gerd* “she Gerd”, *a/hu/ho mor* “she mother”, *n/han far* “he father”, and are used of people that are personally known to SAP. The other set constitutes demonstrative markers which are identical to the set of 3^rd^ person singular personal pronouns, e.g., *hu/hun Berit* “she Berit” (feminine), and *han Ola* “he Ola” (masculine). These demonstratives, labelled “psychological distal demonstratives” (PDD), are used with specific reference to human or human-like nouns (e.g., pets). PPD and PDD are phonologically, semantically, pragmatically, and syntactically distinct, with a higher degree of grammaticalization in PPD and usage reflecting the notion of psychological proximity vs. distance: while the proximal set is used with first names and family relations, the distal set is used with all kinds of nouns denoting humans. In Johannessen’s analysis, PDD signals social distance from speaker and/or addressee towards the referent, connoting the speaker’s negative attitudes for this individual and excluding the possibility of personal acquaintance, while simultaneously indicating that the referent is a specific individual. The use of PDD is categorically conditioned by its specificity; Johannessen (2008, p. 167) states that “The PDD must refer to something specific, never hypothetical or non-existing.” Specificity distinguishes them from regular definite articles, while also interacting with definiteness in interesting ways. This description is in line with the rank of names and demonstratives in the intermediate section of PH: not as proximal as SAP, but still more proximal than the low right end of PH.

A related case is the German usage of definite articles with personal names (*die Gisela,* feminine; *der Jonas*, masculine; Patterson and Schumacher, 2021). A case from noun classes corroborates that personal relationships and individuation are closely connected concepts: while the Setswana noun class 1/2 is reserved for only and most personal nouns, class 1a/2a comprises all personal names, kinship terms and personified animals (Tsonope, 1988, p. 40). The former class represents the Ego, the latter the Ego’s immediate environment.

The reason why kinship terms and uniquely identifying labels like names, epithets, titles, and psychological demonstrative pronouns rank above human nouns when they indeed reference humans, cannot be explained by pointing to “animacy.” Nor has it been explained why these terms associate closely with definiteness, specificity, and demonstratives.

#### The sixth conundrum: if animacy underlies prominence, how can definiteness and specificity be independent drivers of prominence hierarchies?

Surprisingly, whereas PH in some languages respond to animacy alone, other languages are sensitive exclusively to definiteness or specificity (see Figure 2). This means that not just animacy and humanness, but definiteness and specificity as well can independently drive DOM effects. In person-sensitive verbal agreement languages, definiteness plays an essential part; e.g., in Hungarian, agreement with subject is marked on the verb, but objects are only agreement marked if they are definite (Kiss, 2017). If PH is truly based on animacy, aspects like definiteness and specificity are left unexplained. We believe the answer to this could be concreteness and detail associated with proximal construal as it is documented in CLT. Grammatical behavior itself bears witness of this; individuation is a more prevalent feature in pronouns than in nouns and clusters with definiteness and number. For example, number distinctions can be present on pronouns, including 3^rd^ person, that are not present with common nouns, as in Mandarin: Pronouns for 3^rd^ person are differentiated for plurality, e.g., 3^rd^ singular *tā* “he/she/it” vs. 3^rd^ plural, *tāmen* “they”, but not for common nouns, e.g., *shū* “book/books” (Croft, 1990, p. 111; Li and Thompson, 1981, p. 13).

We believe the answer to conundrums 4–6 is that these are instance of individualization and narrow reference, which matches the predictions and experimental findings of CLT that, in proximal construal, people visualize concrete exemplars and construct narrower categories with fewer members than in distal level construal (see Table 1).

### Fronting mechanisms

Various linguistic devices share a formal marking with entities high in PH but has not been linked to these hierarchies in linguistics. Fronting mechanisms refer to various syntactic processes that move an element from its typical position to the beginning of a sentence or clause for various cognitive and communicative purposes, such as emphasis and focus, topic marking, and creating contrast or cohesion in discourse. A shared function of fronting may be to make certain elements more cognitively accessible or easier to process by placing them in a prominent position, potentially reducing cognitive load and facilitating attention.

#### The seventh conundrum: why does one “empathize with” inanimate discourse topics but not with inanimate direct objects?

The “Discourse Topic Empathy Hierarchy” was proposed in Kuno and Kaburaki (1977) and Kuno (1976, p. 267): “It is easier for the speaker to empathize with an object (e.g., a person) which he has been talking about than with an object that he has introduced anew into the discourse: Discourse-Anaphoric > Discourse-Nonanaphoric.” Similarly, in attempting to bridge topic-worthiness and agentivity, Payne (1997, p. 151) invokes the concept of empathy (see also Kuno, 1976) to explain why certain topics are favored in human conversation, see 9):

9) “Humans tend to select as topics entities with whom they empathize, first of all themselves, then the person they are speaking to, then other human beings, and finally the inanimate world. Therefore, morphosyntactic expressions whose function is to refer to topical entities indirectly tend to refer to entities that speakers empathize with.”

In direct/inverse systems, the speaker’s assumed degree of linguistic empathy (Kuno, 1976, 1987; Oshima, 2007a, 2007b) follows a set of constraints: (1) Speech act: the speaker cannot empathize with someone else more than with himself, (2) Topic: the speaker “empathizes” more easily with the discourse topic than non-topic matters, (3) Surface structure: It is easier for the speaker to “empathize with” the referent of the grammatical subject than with referents of other NPs in the sentence, (4) Descriptors: Given descriptor *x* (e.g., Peter) and another descriptor *f(x)* (e.g., Peter’s sister), it is easier to empathize with *x* than with *f(x)*, which is indirectly accessed via the first descriptor. To these generalizations is added a caveat that a single sentence cannot contain logical conflicts in empathy relationships and empathy relationships within a sentence must be consistent (Oshima, 2007a).

The logic of the empathy hierarchy is that proximal participants are considered central to the story and thus higher on an empathy hierarchy than the obviative marked participant within the same sentence (Oshima, 2007a; Kuno and Kaburaki, 1977). However, taking a closer look at these principles, one observes that, on the one hand, inanimate objects are lowest ranking given that one cannot empathize with unliving things, and yet on the other hand, when a non-living entity becomes the topic, it outranks a living, also human, subject, in that respect. Linguistic empathy was recently reiterated as the speaker’s attitude towards and identification with the referents therein (Kann et al., 2023), a concept resembling the social dimension of psychological distance, but which is unintuitive of fronted inanimate objects, and lacking the potentials to be extended into a full model that can explain all aspects of PH, in particular why languages use the same grammatical devices for discourse topicality of inanimate objects as for emotion-based proximity.

Several languages express both types by a unique identifier, e.g., direct/inverse morphology, particles, or case, suggesting the underlying mechanism is basically the same, *viz.* to hold in mind (or draw close) an entity for proximal construal, evident from the following examples. First, Navajo adheres to a strict agency hierarchy (Zúñiga, 2014; Witherspoon, 1977; Drexel, 2014). Deviating from the hierarchy requires the use of the inverse marker *bi-* on the verb when two participants are equal on the PH. While *bi-* in Navajo is a device that marks reversed agency order (DeLancey, 1981), if attached to the postpositional phrase instead of to the verb, it functions as a topicalizing device (Perkins, 1978). Second, the *wa* particle in Japanese is used as a “pragmatic case marker” for participants in the clause that possess the highest degree of inherent topicality, but also for the attention-evoking function of contrast (Maruyama, 2003), and as a topic-marker and a marker for something that is known from previous conversation (Drexel, 2014; Kittilä et al., 2011; Kuno, 1973). Third, while the primary function of ergative case is to mark subjects in transitive sentences (Dixon, 1979), and secondarily to signal agency of inanimate or lower ranked items in DOM languages, the ergative also serve other functions that align with fronting mechanisms in optional ergative systems (Fauconnier, 2011). In the Gooniyandi and Warwa languages, an unexpected discourse topic that is drawn to attention receives ergative marking (McGregor, 1992, 1998, 2006). In Foré, ergative case signals contrastive focus (Donohue and Donohue, 1998, p. 85), and in Umpithamu (Verstraete, 2010) and Waskia (Ross and Paol, 1978, pp. 36–39), ergative simply represents focus. Besides, in Gooniyandi, ergative in commands signifies that a 2^nd^ person agent is accorded special focus or prominence because of surprising or unusual involvement in a process. By contrast, absence of ergative marking on such agents signifies that the addressee agent is obscured or not individuated, because in an avoidance relationship to the speaker (McGregor, 1992). These cases demonstrate that ergative case is not just about agency, but that instead it generally signals a proximal-level construal.

Instead of interpreting such devices as signals of animacy or topicality in the traditional sense, they can be viewed as attention shifters from a default egocentric viewpoint to a reference point—a topic—intently chosen by the speaker in the here-and-now moment. In the CLT framework this would amount to the same thing, since a topic is what is being talked about. In other words, topicalization is to bring an entity into the speech-act participants’ immediate sphere of attention. Topicality infers that the referent of a noun phrase is identifiable by the hearer, creating a sense of “aboutness” (Reinhart, 1981, p. 5) and implicitly shared knowledge (Gundel, 1985, p. 92): “the topic of a speech act will normally be some entity that is already familiar to both speaker and addressee.” Topics are time-sensitive, “what is of current interest or concern” (Strawson, 1964, p. 104); consequently, a topical referent is something that at a certain moment in time presides SAP’s attention and as a result, is attributed salience, or cognitive prominence. Kaiser (2009, p. 335) links salience to attention, stating that anaphoric pronouns correlate with the most salient entities in discourse, in the sense that these referents are at the center of attention.

An alternative to postulating a caveat to grammatical patterns only applicable to linguistics is to invoke a known cognitive constraint, the scarcity of human attention (Simon, 1971), or the fact that attention can only be fully directed to one target at a time. Scarcity of attention interacts with linguistic information structure in important ways. Both provide a narrow and selective focus (Walker, 2002). Just as attention is selective due to its limited capacity, topics in linguistic structure serve to focus the listener to highlighted information in discourse, helping to direct limited attentional resources efficiently and playing a crucial role in organizing discourse structure (Walker, 2002). In discourse, shifts in topics can be alikened to the reallocation of attentional focus when switching tasks; the linguistic cues signal topic changes to help redirect the listener’s limited attentional resources. Thus, attention necessitates prioritization of information, just as topics in linguistic structure indicate what information is deemed most relevant in a given context. Finally, topic constructions align with the constraints of working memory, presenting information in chunks that can be readily processed and maintained in an active state (Oberauer, 2019; Schmidt and Schmidt, 2001).

Topicality is thus a derivative of attention and how this is implemented in grammar finds parallels in human biology. Attention constantly relocates with moving cognizers within their peripersonal space or the individual’s action radius that is neurally encoded in the human brain’s “body schema”, linked to hands, head, and trunk (Di Pellegrino and Làdavas, 2015). It can be reallocated to items by having human eyes and minds directed at it at will. In linguistics, the first type corresponds to inherent “topic-worthy,” and the latter to “context-imparted” topicality (Payne, 1997). In the unmarked case, an entity higher in PH is preferred as the topic as a function of inherent topicality, but this can be reversed if a low-ranking referent is highlighted as a topic for communicative purposes, requiring linguistic cues that are overtly marked. The answers to an apparent enigmatic multifunctionality of inherent vs. context-imparted topicality lies in the level of construal of topics. Within a CLT model of PH, fronting mechanisms are understood in conjunction with the roles of SAP. Topics are marked as proximal in grammars because they inhabit the minds of speech-act participants and the mental space between them in a here-and-now moment. What part of a linguistic construction is higher on PH, is determined either by situatedness and context when drawn mentally close by joint attention. Also, determined by the omnipresence of conscious cognizers aware of their own animate capacities as potential initiators of action chains, but never at the same time due to limitations of human attention. Note also that the SAP pronouns “I” and “you” shift their reference with who speaks and is who spoken to, placing them firmly within context-dependency.

We suggest that linguistic empathy, topicalization in direct/inverse systems, agency and animacy rankings are all aspects of proximal level construal, the egocentric viewpoint in the here and now. By including in the egocentric reference point not just of the self but also the “here and now”, as proposed in CLT, some formerly misconceived phenomena of prominence hierarchies can make sense; since topics are by definition something that a speaker holds in his or her attention, at the moment of utterance. Empathy emanates from the self *and* results from attention, the act of applying the mind to something in the present moment, and that can potentially evoke feelings towards what is presently attended to, as a secondary function of the immediate attention.

#### The eighth conundrum: why can oblique DOM participants be passivized when their homophone obliques cannot?

Split ergative languages employ ergative for nontypical subjects and accusative for nontypical objects. In so-called oblique DOM languages, however, direct objects with specifications characteristic of higher prominence such as being human, animate, definite, or specific, are instead marked with *oblique* prepositions or cases: dative, genitive, or locative. Elsewhere in the language these oblique cases are used in their primary functions of beneficiary, part/owner of, or place/goal.

In discourse, passive voice can help maintain cohesion by keeping the focus on a consistent topic by highlighting the patient role and suppressing the agent. In nominative-accusative languages, the accusative marked patient object in an active sentence, typically low in animacy and prominence, is raised to subject position, attaining all the typical characteristics of subjects. In oblique DOM languages, however, only direct objects can be fronted to fill the subject position in passive constructions, while other arguments marked with these cases within a certain language, cannot (Irimia, 2023). For example, Spanish marks a human direct object with a dative preposition *a*, while inanimate direct objects are unmarked. While the dative DOM can convert to a subject in a passive sentence, the same operation for an ordinary indirect object, with the exact same dative case marking, is ungrammatical, see 10) and 11); examples from Irimia (2023).

10) Veo      **a**     la     mujer/(*a)
       See.1SG     DAT=DOM     DEF.F.SG     woman/DAT=DOM
       la         casa.
       DEF.F.SG. house
      ‘I see the woman/the house.’
       La    mujer/la    casa    fue    vista
       DEF.F.SGwoman/DEF.F.SGhouse was seen.F.SG
       ‘The woman/the house was seen.’
11) Le     doy     el     libro
     CL.3SG.DAT     give.1SG     DEF.M.SG    book
     **a**     la     mujer.
     DAT     DEF.F.SG     woman
     ‘I give the book to the woman.’
     *La     mujer     fue      dada/dado     el     libro.
     DEF.F.SG     woman   was   given.F.SG./M.SG   DEF.M.SG   book
     ‘The woman was given the book.’

The prevailing understanding for this difference in grammatical behavior is that “Oblique DOM is not an oblique syntactically” (Irimia, 2023). However, to analyze this purely in syntactic terms masks the choice of an oblique case for DOM, as well as the connection to other usages of that case within the language. Instead, semantic extension leading to polysemy of these oblique cases to DOM may reveal that case assignment was not made at random. For example, the DOM marker *pe* in Romanian likely evolved from embedded topics and were polysemous with the goal/locative/topical usages *for*, *on*, *concerning*, and *about* in Old Romanian (Onea and Mardale, 2020, p. 362). These usages are lost in modern Romanian and instead developed the values “human” and “definite” required for DOM. The authors point out that “different languages tend to exhibit the same or similar patterns.” Alternatively, to focus on the recipient role in, e.g., *Am cumpărat flori* pe *mama.* “I bought flowers for my mother” would highlight how Romanian overlaps with Spanish DOM.

Thus, in response to this conundrum, we suggest that oblique DOM, as part of cross-linguistic PH phenomena, cannot be explained by syntax alone. Instead, it should be viewed as one of several fronting mechanisms that exploit the semantics of case to associate the characteristics of proximal construal.

### Abstraction

#### The ninth conundrum: why are abstract concepts low in prominence?

In many languages, a broader PH is observed that includes abstract concepts at the lower end of the scale. An expanded hierarchy might look like in 12) (Langacker, 1991a, p. 307):

12) Speaker > hearer > human > animal > plants > physical object > abstract entity

For example, abstract concepts like *hunger*, *health*, or *happiness* are the lowest in prominence in Navajo (Drexel, 2014, p. 9). Blackfoot also makes an interesting case where nouns are classified as animate vs. inanimate/abstract (Ritter, 2014). An intriguing case is Old Romanian where the oblique DOM marker *pe/pre* mandatorily marks proper nouns, which are highly individuated nouns that call for proximal construal, but with the only two recorded exceptions being names of *Hristos* “Christ” and *Dumnezeu* “God” (Onea and Mardale, 2020, p. 359); a likely explanation being that deities were conceived of abstract concepts.

While proximal construal associates with individuation, abstraction associates with group thinking. Bantu noun classes tend to classify abstract concepts with collective nouns, e.g., Setswana (Tsonope, 1988, p. 40). In many nominal classifier systems, insects are classified as inanimate: Indonesian (*kaki*), Lahu (*mà*), Vietnamese (*cai*), or as abstract: Fulfulde (*ngu*) (Lobben et al., 2020, supplement). The gradience here suggests that absence of individuation better explains these collocations than animacy.

To sculpt abstract concepts into a grid made for concrete objects might not be very informative of their constitution or explain why they are the lowest category in PHs. Curiously, very little research has gone into possible reasons for abstract concepts being low in prominence. Generally, the animacy account has been assumed but never substantiated with abstract concepts. For example, Navajo abstract concepts are labelled “incorporeal inanimates”, contrasting with “corporeal inanimates” (Witherspoon, 1977; Perkins, 1978).

In a model of PH within CLT, there is no need for *ad hoc* assumptions of inanimacy in abstract concepts. Instead, abstraction inhabits the far end of hierarchies as a function of psychological distance. Soderberg et al. (2015) review the theoretical rationale for expecting a link between psychological distance and abstraction and provide multiple experiments testing this link. The effect of distance was significant, produced medium-sized effects on construal-level, and was similar across different types of psychological distance: temporal, spatial, social, and hypothetical, supporting CLT’s central prediction that variation along any dimension of psychological distance will influence level of abstraction.

### Cultural flexibility and variance

#### The tenth conundrum: why does linguistic categorization not comply with biology?

Animacy-sensitive DOM may respond to the human category while extending beyond, or retracting within, the human category “in ways that are clearly culturally determined” (Aissen, 2003, p. 456). For example, Yiddish marks direct objects differently depending on humanness, but mandatory case marking is restricted to a set of masculine nouns that denote humans culturally defined as “worthy of respect”: *grandfather*, *teacher*, or an ethnic group member. Older feminine relatives are only optionally case-marked; e.g., *grandmother*, *mother, aunt*. Splitting of the human group can result in categorizing people as non-human. The Marind of Papua New Guinea assign women to animal classes while men inhabit the human class. While such examples take a narrower view to what should be included within the egocentric sphere, there are also examples of the opposite: Ritharngu, an Australian language, extends case-marked objects beyond the human category to include so-called “higher animals” (e.g., kangaroos, dogs, and emus). Animals considered lower on the hierarchy, like fish and raccoons, are not case-marked (Heath, 1980). Moreover, case-marking may leak across the animate-inanimate boundary; in Bayungo, all animate-referring objects are case-marked, including humans, but in addition the two inanimate nouns of *meat* and *vegetable food* (Austin, 1981). This narrow selection of inanimate, case-marked, DOM nouns is unlikely to be random as these nouns reference biological material for consumption and therefore associate with human bodies.

Divisions into “higher” vs. “lower” animals can affect individuation as well. In Tiwi, higher animals are number-marked like humans, while lower animals and inanimates are unspecified for number (Haspelmath, 2013). Further, in Manam, dual and paucal noun forms are used only for humans and “higher animal,” in line with the typological tendency that unmarked grammatical categories display more values than marked ones (Croft, 1990, p. 78). What counts as a higher animal in DOM, however, may come down to whether the animal is domestic or wild, discounting the actual species. Humans are always considered “higher animals,” but pigs, dogs, fowl, and goats only when domesticated (Lichtenberk, 1983, pp. 110, 256). The same kind of ranking between domestic and wild animals is found in Navajo (Drexel, 2014; Perkins, 1978). Although it seems to matter how humans interact with animals, taxa matter towards extremes in natural size and individuation feasibility; the Navajo PH ranks insects lower than “small animals” but higher than natural forces > plants/inanimate objects (Lockwood and Macaulay, 2012).

**Figure 5:**
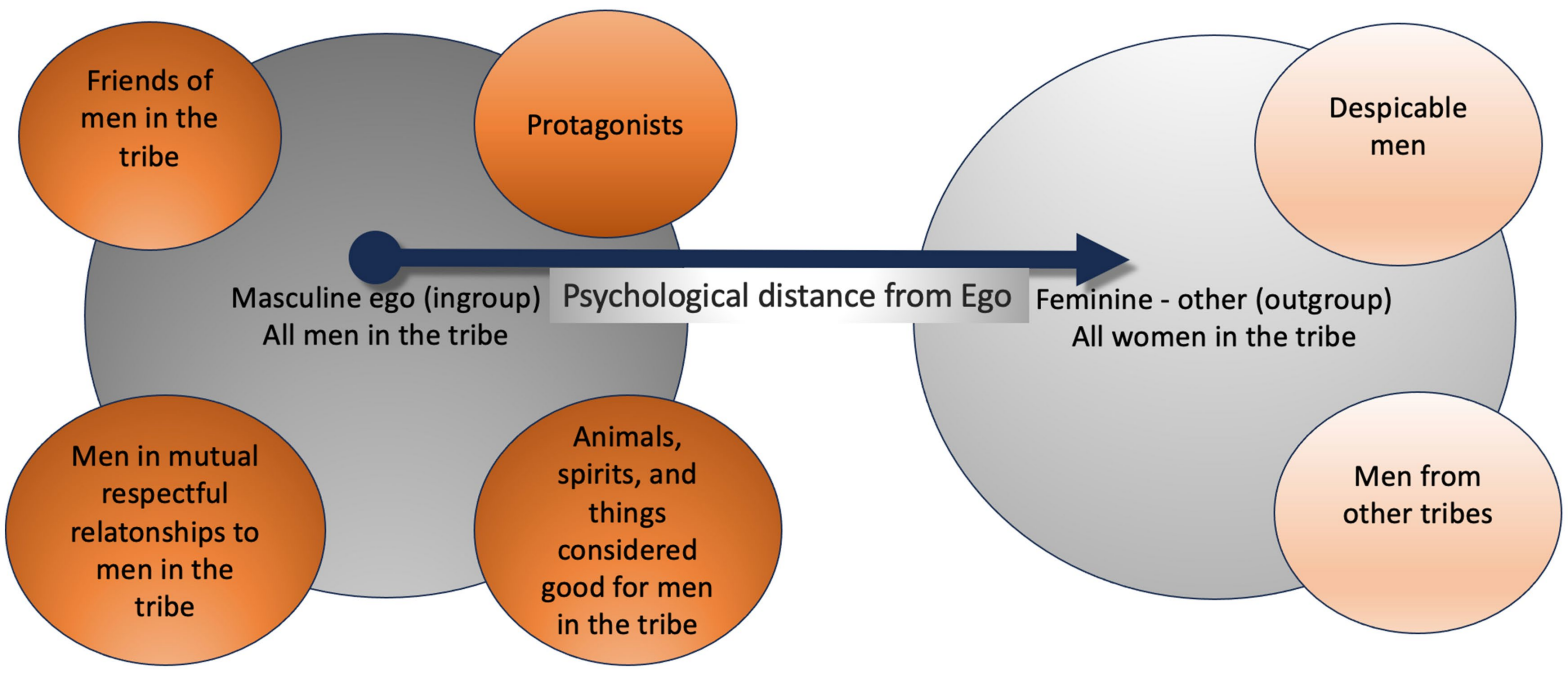
Prototypical category structure in Lokono explained in terms of psychological distance. The value in the large circle is the prototype, and the smaller circles represent the semantic extensions, defined from the viewpoint of a culturally dominant ego.

Divisions of biological sex are largely clear-cut but, in Lokono, grammatical gender seems to follow in-group versus out-group distinctions reminiscent of the PH egocentric systems. Masculine gender is applied to all men within the Lokono tribe, unless they are despised, as well as all things that one considers positive, even animals, things, and spirits (if they are thought to be good or are protagonists within a story). Men of other tribes can be referred to with masculine gender if they are friends of the speaker or in a mutual respectful relationship to him. Feminine gender, by contrast, apart from being used of women within the tribe, are used for despicable men, and men from other tribes (Aikhenvald, 2000, p. 262), see Figure 5. This pans out even more clearly in the Brazilian language Jarawara, where a masculine nominal class can be used selectively to revere women. Conversely, in Amharic culture, feminine gender is applied to men as a marker of respect. Evidently, the prototype from which psychological distance is measured is male in the former two cases, and female in the latter. Apparently, the differences in cut-off points along prominence scales are grounded in construals defined by the culturally dominant supplier of premises and social values that speech societies make within each culture.

Psychological distance can explain these mismatches between culture and biology in linguistic categories, e.g., why some human classifiers do not align with biological distinctions of humans like male and female, why external ethnicities are sometimes excluded from human classification, why domestic animals are ranked higher in PH than wild animals within the same species, and why grammars treat pets different from other animals. Just as the semantic scales in PH and noun categorization are gradient, the conception of psychological distance is continuous with no fixed or predefined cut-off points.

Yet, the clearest evidence of psychological distance impetus comes from cognitive neuroscience. In human brains, ventral visual areas are topographically organized along a gradient scale analogue to linguistic animacy scales; the neural population that respond to face perception is located adjacent to that of primates, birds occupy an area in between the primate and the insect areas, which again border on the regions for inanimate entities (Connolly et al., 2012). A similar topography exists in the monkey (*makaka mulatta*) brain; for example, within face responding neurons, distinct clusters activated primate and non-primate (goat/horse/dog) faces, and primate faces activated separate clusters for humans and monkeys (Kiani et al., 2007). In other words, primates may be innately endowed with a neural grid of animacy gradience and, in humans, this gets expressed in most languages’ structures. Knowing this, one cannot disregard the parallel between the neuroscientific finding that brain regions responding to dogs’ faces were closer to human faces in the human brain’s representational space than, for example, the less familiar but genealogically closer monkeys (Connolly et al., 2011). Moreover, the grammatical status of pets and domestic animals overlaps with that of humans. Since monkeys do not keep dogs as pets, their brains may express the “original” topography.

PH and psychological distance are both *malleable* concepts, as evidenced by cross-linguistic variations in grammatical structures. However, it is important to remember that it is the brain’s neural network that possesses this malleability or plasticity. Evidently, proximal construal overrules genealogical and phylogenetic information and may produce lasting effects on the brain, and in turn, on grammar. By consequence, if there is evidence that peoples’ relationships to their pets can affect brain organization, there are strong reasons to believe that men’s culturally determined relationships to women, and ingroups’ relationships to outgroups, also affect their brains, and in turn, their grammars. All this makes psychological distance a very real-world phenomenon, embodied in the human brain.

## Merge: where linguistics meets psychology

### Cross-field correspondences in construal levels

As discussed in the preceding, the common ground between linguistic prominence and construal levels resulting from psychological distance are multifold. The most conspicuous overlap occurs with the egocentric viewpoint. In taking an integrative approach to linguistics, all the properties associated with the Ego in its own understanding of itself becomes available to characterize the proximal viewpoint. Agency, animacy, empathy become part of a linguistic expression’s prominence as manifestations of the most proximal construal level in any linguistic proposition, whether a 1^st^ or 3^rd^ person pronoun, or an animate noun. At close range, the Ego observes details, individuals, and is at the mercy of the immediate context. Topics are part of this context because they are at the center of current attention in speech-act participants’ minds. When immersed in immediate context, people tend to think about exemplars rather than categories of things; hence, items are definite and specific, and at the social level, personal relationships are one-to-one. This results in narrow categories with either just one or a limited number of referents. In prominence hierarchies, these narrower categories manifest as ranked personal pronouns, personal names, titles, demonstrative personal pronouns, and kinship terms. Although kinship terms are words with generalized meanings, when in use they reference one individual at a time. CLT is resourceful to linguistics in throwing new light on fronting mechanisms. The use of common grammatical marking in several apparently disparate grammatical functions such as topicality, focus, and agency suggests conceptual unity. By incorporating linguistic prominence under CLT’s notion of proximal level construal, these devices will receive a unitary account. As a consequence, the formerly proposed subhierarchies of PH in 1a-d) above become superfluous and can be replaced by *one* hierarchy. Table 2 summarizes the parallels between PH and CLT at the proximal construal level.

**Table 2:** Parallel structural characteristics of proximal construal in CLT and PH.

Proximal construal level characteristics in psychology	Proximal features in prominence hierarchies in languages
Concrete	Concrete
First person perspective	1st person pronoun, may include both SAP
Social ingroup, emotionally close/positive relations (e.g., friends)	Kin, the other SAP, tribal ingroup, dominant gender; domestic animals and pets
Exemplars or narrow categories	Singular pronouns, kinship terms, personal names
Individualized information, details	Personal names; dual, paucal and plural forms
Specific behaviors	Specific/definite referents, including pronouns
Contextualized, situation-specific demands, situational information	Context-imparted topics; pronominal referents vary with context (who speaks, textual context)
Goal irrelevant	Grammatical subject, intransitive actions
Feeling of elevated power	Strength of agency

The CLT variables are sampled from Soderberg et al. (2015) and Trope and Liberman (2003).

In thought and language, the third person perspective brings about different effects on cognition and grammar than first person perspective. While PH prioritize first over third person, considering them more prototypical agents than third person participants, in thought, first-person perspective causes more detailed recalls about events, while third person perspectives bring about more global, dispositional, less detailed, and abstract qualities of the same events. Social distance towards outgroups is related to negative emotions in psychology as well as in language, the latter conspicuous in nominal classifiers. In psychology, social outgroups are associated with structured, predictable, and abstract features, and more homogenous than ingroups, while in language, outgroups are classified with categories further to the abstract, lower end of PH.

In CLT and PH, as the psychological distance from Ego increases, categories become wider and more general, although heterogenous in real life. The divisions along animal species and heterogeneity of inanimate objects are disregarded, unless drawn to attention and supplied with the proximal construal tokens of individuation and concreteness. As the scope widens, categories become more inclusive but also abstract, in line with the functioning of taxonomic systems: the level of abstraction and complexity of features are inversely correlated; superordinate concepts contain more referents but are captured in terms of fewer features, and vice versa (Rosch and Lloyd, 1978). Thus, the most superordinate concepts are also the most abstract. PH in linguistics differ, however, in including in abstract concepts that are intangible (deities, emotions, and sensations), however, this may be a matter of semantic extension in linguistic systems.

Table 3 sums up the properties of distal level construal in language and psychology.

**Table 3:** Parallel structural characteristics of distal construal in CLT and PH.

Distal construal level characteristics in psychology	Distal features in prominence hierarchies in languages
Abstract	Abstract
Third person perspective	3^rd^ person pronouns
Social outgroup; emotionally distant/negative relations (e.g., enemy)	Other tribes and genders; wild animals and game
Broad categories; broad traits	Superordinate categories, e.g., ‘animals’, ‘inanimate objects’; lack of individuation, e.g., insects = abstract
Superordinate; aggregate and dispositional information, primary features	Superordinate ‘animal’ and ‘inanimate objects’; fewer and only essential semantic features of superordinate categories
Decontextualized	Word reference is more independent of situated context (contrast pronouns which have reference variable with context to nouns)
Goal relevant	Direct objects are targets in transitive actions
Overarching goals	Inanimate objects are goals in transitive actions

The CLT variables are sampled from Soderberg et al. (2015) and Trope and Liberman (2003).

### The mental timeline in transitive trajectories

Temporal distance in transitive sentences is the time elapsed from when an agent initiates an action to the impact this has on the direct object. We speak here of mental timelines (Arzy et al., 2009; Oliveri et al., 2009; Bonato et al., 2012; Corballis, 2009). In mental representational space, time can be stretched or compressed; e.g., verbs can reference processes or punctual events, which have vast effects on grammar (Vendler, 1957). The idea of traversing temporal distance is not new to linguistics. Cognitive grammar imagines the trajectory in transitive sentences to be a mental timeline (e.g., Langacker, 1987, pp. 402–403), and cognitive grammar linguists analyzed spatial and temporal expressions with reference to mental paths (Langacker, 2017, p. 178), and conceived time (Langacker, 1991b, p. 150). Conceptual archetypes and image schemas describe events as trajectory-landmark relationships. In a transitive sentence, the subject (typically an agent) is conceptualized as the (moving) trajectory, while the object is the (stationary) landmark. The interaction between these two entities is mapped onto the mental timeline. Recently, temporospatial construals of events were corroborated by the theory of cognitive spacetime (Stocker, 2014), supported by linguistic analysis and substantial experimental evidence, e.g., from eye tracking experiments (Demarais and Cohen, 1998). Thus, while more research could be useful specifically with regard to the PH and spatiotemporal dimensions of psychological distance, there is already ample independent support for the idea that transitivity is mentally represented along temporospatial domains.

## Discussion and conclusion

We aimed to provide a deeper understanding of prominence in hierarchies by taking as starting point the prototypical transitive sentence, defined as typical human-to-inanimate object interactions. We then explained markedness phenomena as deviations from prototypes, stressing the fact that prototypicality as well as markedness reversal are commonly motivated by extralinguistic factors. We identified the transitive sentence with a minimum of two participants as the cognitive unit where prominence occurs, taking into consideration that, while prominence operates within syntagmatic relations (i.e., within a sentence), markedness happens within paradigmatic relations (i.e., between sentences). Deviations from the prototypical sentence arise because other clausal participants compete with the subject for prominence; e.g., topics or human/animate direct objects that are deemed psychologically more proximal to a cognizing Ego. We identified the tokens for prominence as individuation and concreteness, measured in psychological distance from the vantage point of this Ego. The same kind of distance explains lack of individuation in prototypical direct objects.

What sets our analysis apart from other prominence analyses is that lower-ranked categories are accounted for, including abstract categories of nouns at the lowest end of PH. Direct objects are prototypically wider and more general, less individuated, and increasingly abstract *because* they are construed at psychologically distal levels. In our analysis, all fronting mechanisms are grammars’ devices to change a distal level entity to proximal level construal. We see the signs of this in how some grammars treat inverse constructions, topics and focus alike. While the points above constitute our main findings, we relied heavily on the empirical and theoretical work carried out for decades within CLT that temporal, spatial, social, and factual distance is in fact perceived and conceived of in the human mind as *one* measure of distance. This framework allowed us to treat the transitive space–time trajectory emanating from the agent as forged with social distance. While former linguistic analyses have hypothesized that emotional distance might govern prominence constructions, the unification of all these dimensions of psychological distance was in fact the crucial aspect. It scaffolded the present proposal that transitivity itself imposes a temporospatial distance concurrent with the social distance. A serendipitous finding was that gradience in differentially marked objects matched gradience in semantic category structure in noun categorization. By applying the Ego vs. Other psychological distance, we could identify motivations for biology/linguistic category mismatches, and explain, e.g., that categorizing insects as “inanimate” is likely due to lack of individuation rather than misconceptions that insects are not “living things.”

### Prior research

Our proposal differs from former proposals in several respects. Linguists have tried to resolve the puzzle of differential object marking in various ways, including asking “why and how DOM would arise in the first place” (Onea and Mardale, 2020). While recognizing the challenges in finding conclusive answers, we notice that these previously offered solutions remain within linguistic structure and behavior. For example, the idea that DOM arises because “the animacy and referentiality scales are good indicators of prototypical subjects,” or “are indexed for their properties on general notions such as *affectedness*, *transitivity* or *animacy*” (Onea and Mardale, 2020, p. 352). In contrast, we have ventured the inclusion of linguistic prominence within general cognition, which appears to be a radically novel way of thinking about prominence. Nevertheless, we made use of previous analyses along this path since many were integral to and compatible with our proposal. In particular, the notions of egocentricity (Gardelle and Sorlin, 2018), viewpoint and attention flow (DeLancey, 1981), the empathy hierarchy (Kuno and Kaburaki, 1977; DeLancey, 1981; Langacker, 1991a; Oshima, 2007a; Matthews, 2007), counting in the analysis of empathy as a radial schema with the self in the center (Yamamoto, 1999), and finally the innumerable accounts on agency and animacy, often seen as inseparable concepts (Yamamoto, 2006, p. 29).

Where our account radically differs from some other proposals is in their practice and belief that languages are in essence autonomous systems that cannot only be described but also explained by mechanisms exclusive to language (e.g., “modularity of mind,” see Fodor, 1985 and “parallel architecture,” see Jackendoff and Audring, 2019). In our view, instead of being endowed with a special, isolated language faculty, humans possess a language faculty that is deeply intertwined with and building on general cognitive processes, making it an integral part of human cognition. Indeed, “separatists” need to explain the remarkable parallels between prominence phenomena and proximal-distal construal. Much previous meticulous work by linguists (e.g., Aissen, 2003; Irimia, 2023; Starke, 2017; Caha, 2009) may not suffice to fully explain prominence phenomena, as these formalisms may stay at descriptive levels. Thus, our goal is not restricted to *how* languages may be constructed but extends to *why* state of affairs is the way they are.

Our proposal also differs from others in assigning a pivotal role to cultural differences regarding the makeup of linguistic categories. Cultural biases in prominence hierarchies confirms the egocentric perspective. Contrary to Gardelle and Sorlin (2018, p. 134) that “despite an obviously cultural basis, the notion of Animacy Hierarchy appears to be restricted to linguistics; it does not seem to be used, for instance, in sociology, anthropology or philosophy,” we have shown instead that this perspective is indeed what influences the egocentric viewpoint in PH. Given the role of empathy and egocentric perspective in the prominence hierarchy, it is not surprising that cultural differences in social organization appear to influence the grammatical systems, culture here being understood as “collective patterns of thought within a speech society” (Enfield, 2002).

The way in which language systems can co-vary with the cultural belief systems of their speakers has been discussed since the appearance of linguistic relativism via the descriptions offered in Enfield (2002). The idea that cognitive systems may underlie linguistic systems in a culture-specific way, was first elaborated by Wierzbicka (1979), who coined the term “ethnosyntax,” suggesting that “every language embodies in its very structure a certain world view.” Importantly, she went a step further from Sapir-Whorfism by stating that language does more than to code for culture-based semantic cultural content, but code culture-based grammars as well. This approach has been deemed relevant to aspects of the animacy hierarchy (Drexel, 2014). Specifically, ethnosyntax refers to “the study of connections between the cultural knowledge, attitudes, and practices of speakers, and the morphosyntactic resources that employ in speech” (Enfield, 2002, p. 4). Indeed, some of linguists’ challenges “that beset grammatical theory derive from trying to analyze native speakers’ linguistic knowledge as a self-contained system”; as Keesing (1979) pointed out after years of fieldwork on the Solomon Islands with the Mailaita language.

Although linguists have hinted at cultural explanations for linguistic behaviors described as “cultural flexibility” above, no explanation has been offered that is valid cross-linguistically. For example, Aissen (2003, p. 457) suggests that there are two ways to analyze these cases; either are the categories HUMAN, ANIMATE, and INANIMATE understood differently in particular languages, or there is further language-particular ranking within these ontological categories. Neither of these proposals offer a real explanation beyond the descriptive level. Crucially, none of them provided a comprehensive or *unitary* understanding to all aspects of prominence with reference to universal principles of human cognition, while at the same time allowing for considerable cultural flexibility. Finally, we missed an account that went beyond the mere descriptive level to elaborate on *why* prominence hierarchies look the way they do, how they arise and are maintained in grammars.

### Predictions

Several potential predictions followed from integrating PH and nominal categorization within CLT. One was that as more languages are investigated, prominence and markedness phenomena already described within linguistics will lend themselves to the characteristics of proximal vs. distal level construals, marked with the set of resources for marking prominence existing in the individual language. Perhaps more important was the prediction that prominence in language are concurrent with peoples’ perceptions of prominence outside of language, including their cultural experience and social organization. Another bold prediction was that cross-linguistic variation in PH cut-off points respond to cultural beliefs and practices, or even that within individual languages that possess both type of phenomena, semantic classification in nominal classifier systems and PH will concur. All this can be tested further and should set the ground for a whole new paradigm of research.

### Future research

By aligning linguistic structure with the concept of construal levels modified by psychological distance, we hope has opened up a whole new field for potential new research. One possible direction for future research regards the assumed correspondence between culture and social distance on the one hand, and linguistic structure on the other. This is relevant for nominal categorization as well as prominence hierarchies. As for the question of cultural variance in noun classification, we indicated some “unexpected memberships” that contradicted biological facts and instead followed preferences along gender and ethnicity. We suppose that these are cultural or ethnocentric, defined subconsciously by the dominant social group within societies, although anthropological studies will need to confirm this connection.

Another proposal for future research is to explore the variance in cut-off points along the PH with respect to split case systems. One should note that differences could be motivated by culture or by general cognitive processes. Research on diachronic change shows that the evolutionary paths of DOM may proceed as gradual spreading from the left to the right on animacy and referentiality scales (Onea and Mardale, 2020, p. 351). For example, Lichtenberk (1983, p. 133) predicted that DOM in Manam presently is used for humans/higher animals but is “moving toward a stage where -*di* will be the predominant, if not the only, 3pl object marker.” If this is indeed the case in many languages, the arbitrary cut-off points represent could reflect general tendencies in historical development. The overall variation in categories involved in prominence hierarchies speaks against this being the only cause, however; more likely the causes could be mixed.

Finally, there is a dire need for more research on how abstract concepts behave within PH, including the description of multiple typically varied languages, and a semantic characterization of such abstract concepts.

## Conclusion

Psychological distance is able to explain categorization in aspects of noun classification as well as in PH. Thus, we have proposed an analysis valid for two independent aspects of “animacy” in human grammars by reference to one and the same mechanism. Psychological distance subsumes all the features of PH: agency, emotion, cognition, animacy, and abstraction, and makes it highly plausible that the special features of prominence hierarchies arose out of how the human mind in general cognizes about proximal and distal events and objects; that is, from a subjective perspective towards the external world. All of these individual *manifestations* of prominence can be understood in terms of the overarching notion of psychological distance.

## Data Availability

The original contributions presented in the study are included in the article/supplementary material, further inquiries can be directed to the corresponding author.
